# The Gain-of-Function p53 R248W Mutant Promotes Migration by STAT3 Deregulation in Human Pancreatic Cancer Cells

**DOI:** 10.3389/fonc.2021.642603

**Published:** 2021-06-11

**Authors:** Luisa Klemke, Clara F. Fehlau, Nadine Winkler, Felicia Toboll, Shiv K. Singh, Ute M. Moll, Ramona Schulz-Heddergott

**Affiliations:** ^1^ Institute of Molecular Oncology, University Medical Center Göttingen, Göttingen, Germany; ^2^ Department of Gastroenterology, Gastrointestinal Oncology and Endocrinology, University Medical Center Göttingen, Göttingen, Germany; ^3^ Department of Pathology, Stony Brook University, Stony Brook, NY, United States

**Keywords:** mutant p53, missense p53 mutant, STAT3, selectivity, specificity, PDAC, GOF, Hsp90

## Abstract

Missense p53 mutations (mutp53) occur in approx. 70% of pancreatic ductal adenocarcinomas (PDAC). Typically, mutp53 proteins are aberrantly stabilized by Hsp90/Hsp70/Hsp40 chaperone complexes. Notably, stabilization is a precondition for specific mutp53 alleles to acquire powerful neomorphic oncogenic gain-of-functions (GOFs) that promote tumor progression in solid cancers mainly by increasing invasion and metastasis. In colorectal cancer (CRC), we recently established that the common hotspot mutants mutp53^R248Q^ and mutp53^R248W^ exert GOF activities by constitutively binding to and hyperactivating STAT3. This results in increased proliferation and invasion in an autochthonous CRC mouse model and correlates with poor survival in patients. Comparing a panel of p53 missense mutations in a series of homozygous human PDAC cell lines, we show here that, similar to CRC, the mutp53^R248W^ protein again undergoes a strong Hsp90-mediated stabilization and selectively promotes migration. Highly stabilized mutp53 is degradable by the Hsp90 inhibitors Onalespib and Ganetespib, and correlates with growth suppression, possibly suggesting therapeutic vulnerabilities to target GOF mutp53 proteins in PDAC. In response to mutp53 depletion, only mutp53^R248W^ harboring PDAC cells show STAT3 de-phosphorylation and reduced migration, again suggesting an allele-specific GOF in this cancer entity, similar to CRC. Moreover, mutp53^R248W^ also exhibits the strongest constitutive complex formation with phosphorylated STAT3. The selective mutp53^R248W^ GOF signals through enhancing the STAT3 axis, which was confirmed since targeting STAT3 by knockdown or pharmacological inhibition phenocopied mutp53 depletion and reduced cell viability and migration preferentially in mutp53^R248W^-containing PDAC cells. Our results confirm that mutp53 GOF activities are allele specific and can span across tumor entities.

## Introduction

Already in the early 1990s, the tumor suppressor p53 was coined as ‘guardian of the genome’ (1, 2) and it was known that mutation of the *TP53* gene (tumor protein p53, HGNC:11998) is an essential step in human tumor development (1, 3). Ever since, scientists have tried to understand the influence of the *TP53* status within the mutational landscape in different cancer entities and to investigate the role of different variants in tumorigenic pathways. It became evident that some p53 mutant protein variants do not only abrogate tumor suppressor functions (loss-of-function, LOF) but also gain new tumorigenic functions (gain-of-function, GOF). Given that approx. 70% are missense mutations leading to amino acid substitutions mostly in the DNA binding domain, some alleles are selected and occur at a high frequency, termed hotspots. Most hotspot mutants gain neomorphic tumorigenic functions, particularly in invasion and metastasis of solid tumors (4–9). A key prerequisite for the GOFs of some missense p53 mutants (termed here ‘mutp53’) is protein stabilization through the Hsp90/Hsp70/Hsp40 (heat shock protein 90/70/40) chaperone machinery, resulting in protection from MDM2 (mouse double minute 2) and other E3 ligases and thus proteasomal degradation (5, 10–15).

Due to the heterogeneity of *TP53* point mutations, whose phenotypes in addition are highly dependent on the cellular context, different missense mutants exert different cellular responses (16–18). Thus, it is important to consider the context- and allele-dependent specificity of different mutp53 proteins (16, 19–21). To investigate the mutp53 specificity, different groups have dissected the impact of various mutp53 GOF alleles on tumorigenesis using autochthonous mouse models (22–26) or clinical correlation studies (26–29). Recent results from our group highlight the GOF hotspot mutp53^R248Q/W^ specificity in mouse and human colorectal cancer (CRC). mutp53^R248Q/W^ binds to and deregulates phosphorylated STAT3 (signal transducer and activator of transcription 3) by protecting it from SHP2 phosphatase (*PTPN11*, protein tyrosine phosphatase non-receptor type 11), its major negative regulator. Thus, depletion of mutp53^R248Q/W^ inhibits STAT3 signaling and causes suppression of tumor invasion and proliferation (26). The p53 R248 hotspot is the single most common variant in all *TP53*-altered tumor types occurring in 9% of cases, which translates to about 66,000 newly diagnosed cancer patients in the US per year harboring R248 variants. Of R248 substitutions, over 90% are either Q or W, with similar frequencies (The Cancer Genome Atlas Program – National Cancer Institute).

Here, we asked whether mutp53^R248W^ also exhibits tumor-promoting functions affecting migration in pancreatic ductal adenocarcinoma (PDAC). Note that the *TP53* ^R248Q^ allele is not available in established PDAC lines. PDAC is currently the fourth leading cause of cancer death worldwide with a rapidly ascending trajectory, and the incidence is predicted to increase even further in the future (30, 31). PDAC, which constitutes around 90% of all pancreatic malignancies, is highly aggressive and chemoresistant and still has a dismal 5-year survival rate of only approx. 9% (30, 32–34).

In approx. 70% of PDAC patients, *TP53* undergoes mainly missense mutations (www.cbioportal.org) as a late genetic event at the transition from high grade PanIN dysplasia to invasiveness during pancreatic cancer progression (35, 36). Here, we show in a panel of common human PDAC cell lines harboring different homozygous missense p53 mutants that mutp53 variants differ in their protein stability, with mutp53^R248W^ again accumulating the highest protein levels also in the pancreatic cell context. Importantly, comparing all PDAC lines, only mutp53^R248W^ depletion strongly reduced migration capacity. In support, mutp53^R248W^ specifically showed the strongest binding to phosphorylated STAT3 under baseline and cytokine-stimulated conditions, forming a constitutive mutp53^R248W^-pSTAT3 complex. Only mutp53^R248W^ depletion was able to reduce pSTAT3 levels. Consequently, targeting the tumor-promoting mutp53^R248W^-pSTAT3 complex by pSTAT3 depletion or pharmacological inhibition diminished cell viability and migration in mutp53^R248W^ expressing, but not in mutp53^R273H^ or mutp53^R282W^ expressing PDAC cells. Our results support a GOF function of mutp53^R248W^ in pancreatic cancer cell lines, justifying future investigations in this tumor entity *in vivo.*

## Material and Methods

All materials used and corresponding information are provided as **Supplementary Table 1**.

### Cell Culture

Homozygous mutant human pancreatic cancer cell lines MIA-PACA-2 (mutp53^R248W)^ (DZMS, RRID : CVCL_0428), PANC-1 (mutp53^R273H^) (ATCC, RRID : CVCL_0480), BXPC-3 (mutp53^Y220C^) (ATCC, RRID : CVCL_0186), and PA-TU-8902 (mutp53^C176S^) (DSMZ, RRID : CVCL_1845) were grown in DMEM (Gibco) with 10% FBS (Merck). PA-TU-8988T (mutp53^R282W^) (DSMZ, RRID : CVCL_1847) were grown in DMEM medium with 5% FBS. CAPAN-1 (mutp53^A159V^) (ATCC, RRID : CVCL_0237) were grown in RPMI 1640 (Gibco) with 20% FBS, and L3.6pl cells (truncating frameshift p53 mutation) (37, 38) were grown in RPMI 1640 with 10% FBS. All media were supplemented with Penicillin-Streptomycin (10,000 U/mL, Gibco) and L-Glutamine (Gibco). All cell lines were grown at 37°C at 5% CO_2_ in a humidified atmosphere and tested for mycoplasma contamination on a regular basis (Mycoplasma Detection Kit, Lonza). Cell line authentication certificates are provided as **Supplemental Material**.

### Transfection With siRNA

Depletion of human *TP53* or *STAT3* mRNAs was achieved by siRNA transfection using Lipofectamine™ 3000 (Invitrogen) or Lipofectamine™ 2000 (Invitrogen) transfection reagents. siRNA sequences are listed in supplemental **Supplementary Table 1**. Cells were reverse transfected in 6-well plates (Sarstedt) according to manufacturer guidelines. After 24 h, supernatant was collected and replaced by fresh culture medium. Seventy-two-hour post-transfection cells were harvested for analyses.

### Immunoblot Analysis

Cell lysates were prepared with RIPA buffer containing 20 mM Tris-HCl pH 7.5, 10 mM EDTA, 1% sodium deoxycholate, 150 mM NaCl, 1% Triton X-100, 0.1% SDS, phosphatase inhibitor consisting of 2 mM imidazol, 1 mM sodium orthovanadate and 1 mM sodium fluoride, and cOmplete™ mini protease inhibitor cocktail (Roche). Samples were lysed in RIPA buffer with sonication. Protein concentrations were determined by BCA protein assay (Pierce). Equal amounts of lysates were loaded (15–30 µg) and separated by SDS-polyacrylamide gel electrophoresis followed by transfer onto nitrocellulose membranes (Amersham). After blocking with 5% milk (Roth), membranes were incubated with the following antibodies: HSC70 [B-6] (Santa Cruz), beta-Actin (Abcam), total-AKT [D9E] (Cell Signaling), p53 [DO-1] or HRP-conjugated p53 [DO-1] (Santa Cruz), phospho-Y705 STAT3 [EP2147Y] (Abcam), total STAT3 (Santa Cruz) or total STAT3 [79D7] (Cell Signaling), MDM2 [IF-2] (Calbiochem^®^/Millipore), p21 Waf1/Cip1 [12D1] (Cell Signaling). Primary antibodies were detected with HRP-conjugated secondary antibodies. Signal was developed using Clarity Max™ Western ECL Substrate (BioRad), SuperSignal™ West Femto Maximum Sensitivity Substrate (ThermoFisher Scientific), or Immobilion Western chemiluminescent HRP substrate (Millipore/Merck). For antibody details, see **Supplementary Table 1**.

### Co-Immunoprecipitation

For coIP, cells were lysed in a buffer containing 50 mM Tris-HCl, pH 7.5, 150 mM NaCl, 1% Nonidet™ P40, 10 μM MG-132, phosphatase inhibitor consisting of 2 mM Imidazol, 1 mM sodium orthovanadate, and 1 mM Sodium Fluoride, and cOmplete™ mini protease inhibitor cocktail (Roche), followed by sonication. After centrifugation, samples were precleared with protein G Sepharose (GE Healthcare) and equal amounts of protein were immunoprecipitated using antibodies against total STAT3 (Santa Cruz), phospho-Y705 STAT3 (Abcam), or control IgG antibody (Abcam). Precipitates were analyzed by immunoblotting. For coIPs, p53 was immunoblotted with an HRP-conjugated p53 antibody (Santa Cruz). 5% of each input was used as input control and stained with beta-Actin (Abcam) as loading control. To stimulate STAT3, cells were treated with 50 ng/mL IL-6 or OSM 24 h prior to performing the CoIP.

### Cycloheximide Chase

To evaluate the stability of different mutp53 proteins in the panel of PDAC cell lines, Cycloheximide (CHX) chase experiments were performed. Cells were treated with 40 µg/mL Cycloheximide (Sigma-Aldrich) or ethanol vehicle control for 8 h and 24 h. Protein lysates were prepared with RIPA buffer as described in immunoblot analysis.

### Cell Growth Assay After Hsp90 Inhibition

To investigate HSP90 chaperone dependent stabilization of different mutp53 proteins, cells were treated with Hsp90 ATPase inhibitors Ganetespib (Synta Pharmaceuticals) or Onalespib (Selleckchem). To determine cell confluency, cells were seeded in 96-wells (Corning) and treated with Onalespib or Ganetespib for 24 h. Confluency was determined using the Celigo Imaging Cytometer and the according software (Nexcelom, Software v5.0.0.0).

### Treatment With Cytokines (IL-6, OSM)

To stimulate the STAT3 pathway, cells were seeded in 6-well plates (Sarstedt) and treated with Interleukin-6 (IL-6) or Oncostatin M (OSM 209a.a.) (both from Immunotools) or solvent control for 24 h and analyzed by immunoblots.

### Cell Viability Assay After Stattic Treatment

Cells were seeded in 96-well plates (Corning) and treated with increasing concentrations (0-80 µM) of Stattic or solvent control for 24 h. The CellTiter-Glo^®^ Luminescent Cell Viability Assay (Promega), based on detectable ATP, was performed according to manufacturer’s guidelines. Each biological replicate was measured in triplicates, and viability was calculated relative to the solvent control for each cell line.

### Wound Healing Assay

Twenty-four hours after transfection with siRNAs or scrambled control, cells were incubated in serum-reduced media (1% FBS). Forty-eight hours post transfection, three scratches per well were made with a 1ml pipette tip or 200µl pipette tip as dublicates. Forty-eight hours after scratching, at least five images per scratch were taken, quantified, and averaged per experiment. The degree of wound healing was determined by measuring the scratched area per image using the ‘polygon selection function’ of Image J software. Wound healing rate was measured by averaging each scratch area after 48 h relative to the initial area at 0 h. Biological replicates are defined as independent experiments with cells at different passages and different days. For technical replicates, cells from one experiment were seeded in two different wells (duplicates).

### Transwell Migration Assay

Cells were either transfected with siRNA against TP53 mRNA, STAT3 mRNA or scrambled control. Seven-two hours after siRNA transfection, cells were trypsinized and seeded into transwell inserts (Corning) in serum-reduced media (1% FBS for MIA-PACA-2, PANC-1, BXPC-3 and PA-TU-8902; 0.5% FBS for PA-TU-8988T). Wells (Corning) were filled with the respective complete medium of each cell line. To investigate migration potential upon the STAT3 inhibitor Stattic, cells were seeded in transwell inserts in serum-reduced media. Different concentrations of Stattic or respective control were added to the cells 1-2 h after seeding, allowing cells to settle before treatment. Wells were filled with complete medium. Attempting to induce migration of PA-TU-8902 cells, cells were pre-seeded in 6-well plates (Corning) and pre-treated with 50 ng/mL IL-6 or OSM (Immunotools). After 24 hrs pre-treatment, cells were transferred to transwell inserts, and cytokines were added again.

In the final 24 h after seeding, cells that had migrated to the underside of the membrane were carefully washed with PBS, fixed in ice-cold methanol for 10 min and stained with crystal violet (0.1% in 20% EtOH) for 20 min. After washing, remaining cells inside the insert were removed using a pre-wet Q-tip. The migrated cells were visualized by light microscopy and analyzed using Image J. The migration rate was calculated relative to scrambled siRNA or solvent control, respectively. Biological replicates are defined as independent experiments with cells at different passages and different days. For technical replicates cells from one experiment were seeded in two different transwell inserts (dublicates).

### Analysis of Human Patient TCGA Data

Human genomic data including *TP53* gene mutation and clinical information were downloaded from cBioPortal (www.cbioportal.org). We used cBioportal Pancreatic ductal adenocarcinoma database in this analysis (39, 40). Two datasets were used to detect mutated samples and the clinical data, QCMG, Nature 2016, and TCGA, PanCancer Atlas (41, 42). *TP53* missense mutant group was sampled with *TP53* missense mutations (MS) with indicated amino acid changes, and the *TP53* LOF group was sampled with frameshift (FS) and nonsense (NS) *TP53* mutations. R language (The R Project for Statistical Computing, https://www.r-project.org, version 4.0.2) and the package “survival” were used in the analysis, including calculating log-rank p-value and Kaplan–Meier curves.

### Statistical Analysis

The number of biological and technical replicates (mean ± SEM) is provided in the figure legends. For all experiments, an unpaired Student’s t test (two-sided) was used to calculate p-values.

## Results

### p53 Missense Mutants in Human PDAC Cell Lines Are Stabilized *via* Hsp90

Since different p53 mutants have different conformations and thus different tumorigenic functions that additionally depend on specific cellular/oncogenic context, each allele and tumor type constellation should be considered separately (6, 10, 17, 43). To investigate the allele specificity of mutated *TP53* in pancreatic ductal adenocarcinoma (PDAC), we used homozygous human PDAC cell lines expressing different endogenous p53 hotspot and non-hotspot missense mutants. The panel included CAPAN-1 (p53^A159V^), BXPC-3 (p53^Y220C^), PANC-1 (p53^R273H^), MIA-PACA-2 (p53^R248W^), PA-TU-8902 (p53^C176S^), and PA-TU-8988T (p53^R282W^). L3.6pl harbors a truncating LOF mutation and served as p53null control. Unfortunately, an established PDAC line with a mutated *TP53* ^R^ *^248Q^* allele is not available. The absence of wildtype p53 function was verified in all cases (**Supplementary Figure 1**).

**Figure 1:**
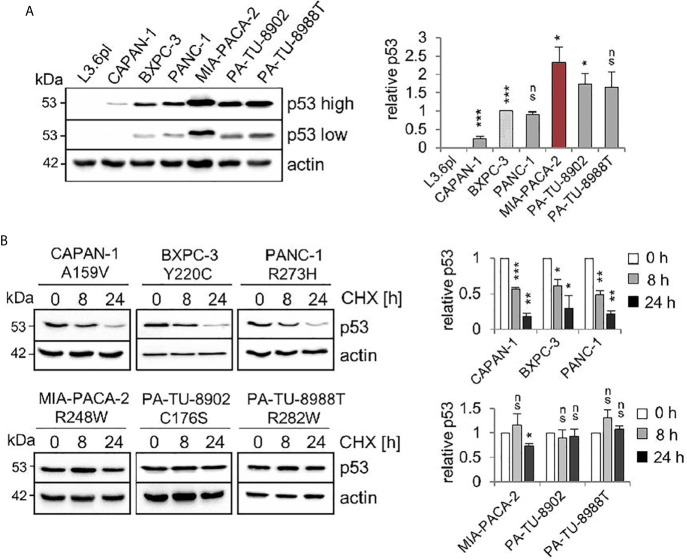
Stabilization of various missense p53 mutants in human PDAC cell lines. **(A)** Six PDAC cell lines harboring various missense mutant p53 variants exhibit differential steady state protein levels. One representative immunoblot analysis out of four is shown. Actin as loading control. ‘p53 high’ and ‘p53 low’ mean exposure time. CAPAN-1 (mutp53^A159V^), BXPC-3 (mutp53^Y220C^), PANC-1 (mutp53^R273H^), MIA-PACA-2 (mutp53^R248W^), PA-TU-8902 (mutp53^C176S^) and PA-TU-8988T (mutp53^R282W^). L3.6pl cells harboring a truncating LOF mutation served as p53 null control. (right) Diagrams represent the means ± SEM of densitometric quantifications of two independent experiments with two technical replicates each (total n = 4 immunoblots), normalized to actin or HSC70 and calculated relative to mutp53 level in BXPC-3 cells (patterned bar). **(B)** Differential half-lives of mutp53 proteins. Cycloheximide (CHX) chase experiment. Cells were treated with CHX for 8 and 24 h or vehicle control (0 h). One representative immunoblot. Actin, loading control. (right) Diagrams represent mutp53 protein levels as means ± SEM of densitometric quantifications of two independent experiments (n = 2), normalized to actin or HSC70. Calculated relative to control treatment (0 h). **(A, B)** Student’s t test. *p ≤ 0.05; **p ≤ 0.01; ***p ≤ 0.001; ns, not significant.

Comparative immunoblot analysis identified the highest steady state protein levels in MIA-PACA-2 cells expressing the R248W mutant (**Figure 1A**). The second highest levels were observed in C176S and R282W harboring PA-TU-8902 and PA-TU-8988T cells, respectively. The lowest level was seen in A159V expressing CAPAN-1 cells (**Figure 1A**). Cycloheximide chase experiments confirmed that the highest p53 steady state levels in cells harboring mutp53^R248W^, mutp53^C176S^, and mutp53^R282W^ were also the most stable proteins with the longest half-lives, while mutant p53 protein with the lowest level (A159V) had the shortest half-life (**Figure 1B**).

**Figure 2:**
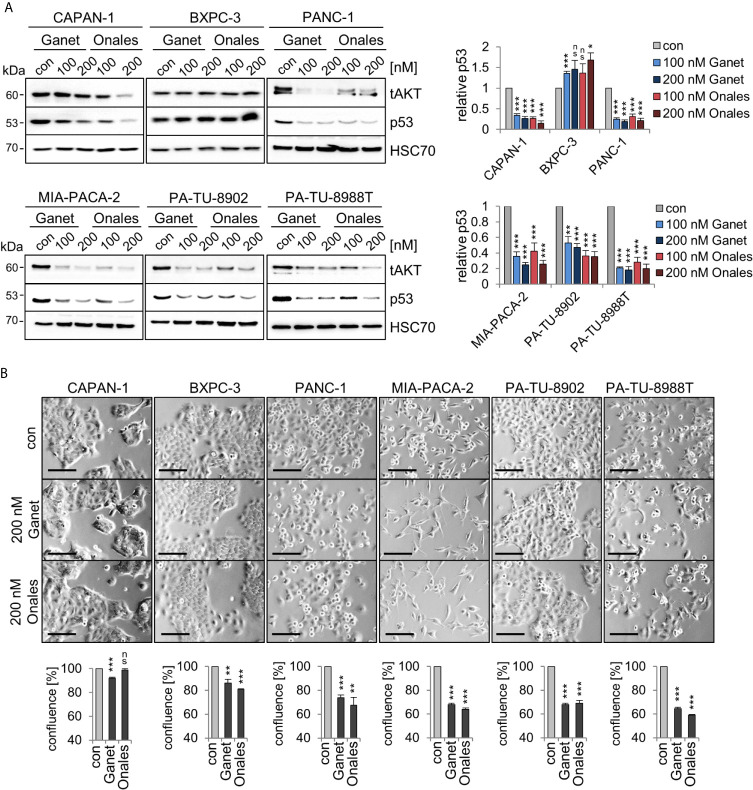
Missense p53 mutants in PDAC cells are stabilized by Hsp90. **(A)** Hsp90-dependent aberrant stabilization of mutp53 proteins in PDAC cell lines. Cells were treated for 24 h with the indicated concentrations of Ganetespib, Onalespib, or DMSO. One representative immunoblot out of three each is presented. HSC70, loading control. Total AKT (‘tAKT’, AKT serine/threonine kinase 1) as well-known Hsp90 client serves as functional control for an Hsp90 inhibition. (right) Diagrams represent the means ± SEM of densitometric quantifications of at least two independent experiments with technical replicates (total n ≥ 3 immunoblots), normalized to HSC70. Calculated relative to control DMSO treatments (con). **(B)** Cell confluence determination. Representative images of cells after treatment with 200 nM Ganetespib, Onalespib, or solvent control for 24 h. Cell confluency was analyzed using a Celigo imaging cytometer. Scale bars, 100 µm. Confluence was calculated relative to their respective DMSO control from n = 3 biological replicates. **(A, B)** Student’s t test. *p ≤ 0.05; **p ≤ 0.01; ***p ≤ 0.001; ns, not significant.

A key prerequisite for the gain-of-function (GOF) of some missense p53 mutants is protein stabilization through the Hsp90 chaperone machinery. Importantly, the clinically relevant Hsp90 inhibitors Ganetespib or Onalespib provide therapeutic selectivity toward tumor epithelial cells but not normal cells, making them attractive for anti-cancer therapies (44). Furthermore, in other cellular contexts such as lymphoma (23), treatment with the Hsp90 inhibitor Ganetespib downregulated mutp53 protein levels. In most PDAC cells, except BXPC-3 cells (**Figure 2A**), Ganetespib or Onalespib also decreased mutp53 protein indicating that mutp53 proteins are mainly stabilized in this context by the Hsp90 chaperone machinery. In line with this, PANC-1, MIA-PACA-2, PA-TU-8902, and PA-TU-8988T cells showed diminished cell growth by about 40%, while the other lines had less reduction (**Figure 2B**). These data reinforce that at least some mutp53 proteins in PDAC might also be targetable with Hsp90 inhibitors.

### The p53^R248W^ Mutant Selectively Promotes Migration in PDAC Cells

**Figure 3:**
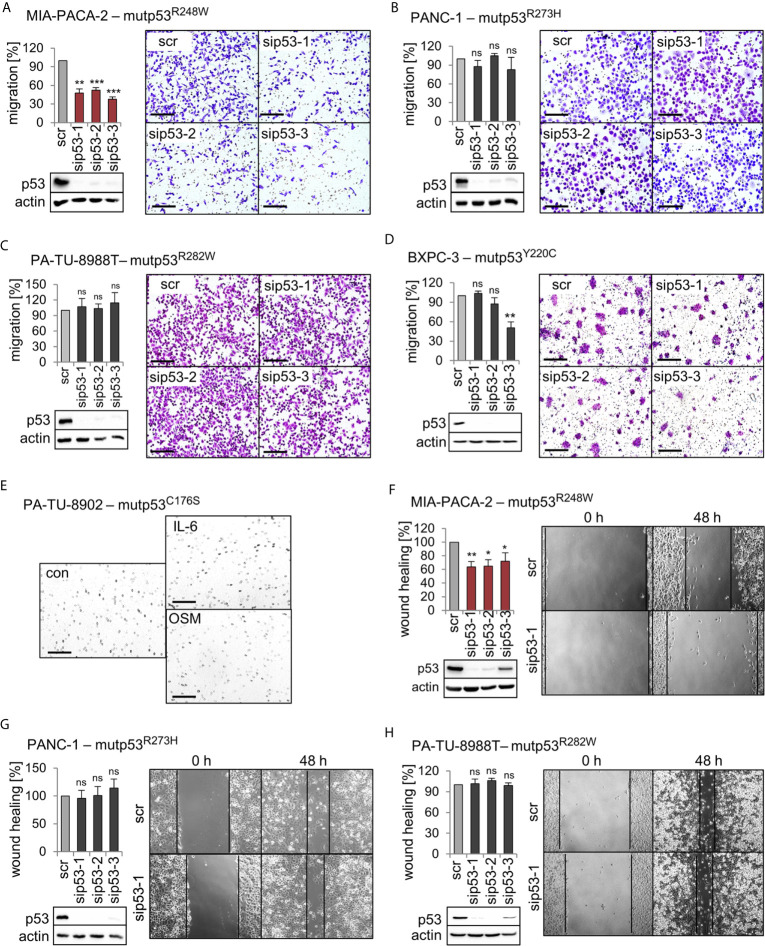
Mutp53^R248W^ selectively promotes migration in PDAC cells. **(A–D)** Transwell migration assays of MIA-PACA-2, PANC-1, PA-TU-8988T, and BXPC-3 cells to evaluate mutp53-dependent migration activity. mutp53 was depleted with three different siRNAs against *TP53* mRNA (sip53 1-3). Seventy-two hours post-transfection with siRNAs, cells were seeded into transwell inserts and migration to the membrane underside was determined after 24 h. MIA-PACA-2 cells: 3 biological replicates (n = 3), PANC-1 cells: 2 biological replicates (n = 2), PA-TU-8988T cells: 3 biological replicates (n = 3), BXPC-3 cells: 3 biological replicates, one with a technical replicate (n = 4). Note, siRNA ‘sip53-3’ reduced migration in BXPC-3 cells might be a consequence of siRNA off-target effects. Migration was calculated relative to scrambled control (scr, set as 100%). Representative images of membrane undersides are shown. Scale bars, 200 µm. Immunoblot analysis verifies knockdown of mutp53. Actin, loading control. **(E)** Transwell migration assay of PA-TU-8902. Representative images of stained transwells after 24 h of migration are shown. To induce migration, cells had been stimulated for 24 h with 50 ng/mL Interleukin-6 (IL-6), Oncostatin M (OSM), or solvent control (con) prior to seeding into inserts, followed by additional cytokine treatment for another 24 h. Gray dots are pores of the membrane. Scale bars, 200 µm. **(F–H)** mutp53-dependent wound healing of MIA-PACA-2, PANC-1, and PA-TU-8988T cells. mutp53 knockdown for 48 h using three different siRNAs (sip53 1-3). Forty-eight hours post-transfection, scratch assays were performed for another 48 h. A minimum of five images were taken and quantified. MIA-PACA-2 cells: 3 biological replicates, 1 out of 3 with a technical replicate (n = 4), PANC-1 cells: 2 biological replicates, 1 out of 2 with a technical replicate (n = 3), PA-TU-8988T cells: 2 technical replicates (n = 2). Wound healing capacity was calculated relative to scrambled control (scr). Representative images after 0 h and 48 h are shown. Solid lines represent edges of the scratch. Immunoblots verify knockdown of mutp53. Actin, loading control. **(A–D, F–H)** Diagrams represent the means ± SEM. Student’s t test. *p ≤ 0.05; **p ≤ 0.01; ***p ≤ 0.001; ns, not significant.

We previously established that a main GOF activity of the mutp53^R248W^ and mutp53^R248Q^ in colorectal cancer compared to p53 null is promotion of cell migration and invasion in tumors *in vivo* and *in vitro* (26). To test whether this is also the case in PDAC, we performed migration assays. Of note, transwell migration assays showed that only siRNA-mediated depletion of mutp53^R248W^ decreased the migration capacity of MIA-PACA-2 cells, while depletion of other alleles failed to do so (**Figures 3A–D**). Interestingly, PA-TU-8988T and PA-TU-8902 cells, which also express high levels of stabilized mutp53^R282W^ or mutp53^C176S^, respectively (**Figures 1A, B**), did not show reduced migration after mutp53 depletion (**Figure 3C**) or were completely unable to migrate through the pores of the transwell membrane (**Figure 3E**). This remained even after treatment of PA-TU-8902 cells with the cytokines Interleukin-6 (IL-6) and Oncostatin M (OSM) (**Figure 3E**), known to induce migration and proliferation in numerous cell types (45–47). This suggests that high mutp53 stabilization per se is a necessary but not sufficient precondition for acquiring a GOF on migration.

To confirm the effects seen in migration assays, three cell lines were further analyzed by wound healing scratch assays. Again, specifically MIA-PACA-2 cells bearing the R248W mutation showed the strongest reduction in wound closing capacity upon mutp53 depletion (**Figures 3F–H**).

### Mutp53^R248W^ Selectively Binds to Phosphorylated STAT3 in PDAC Cells

**Figure 4:**
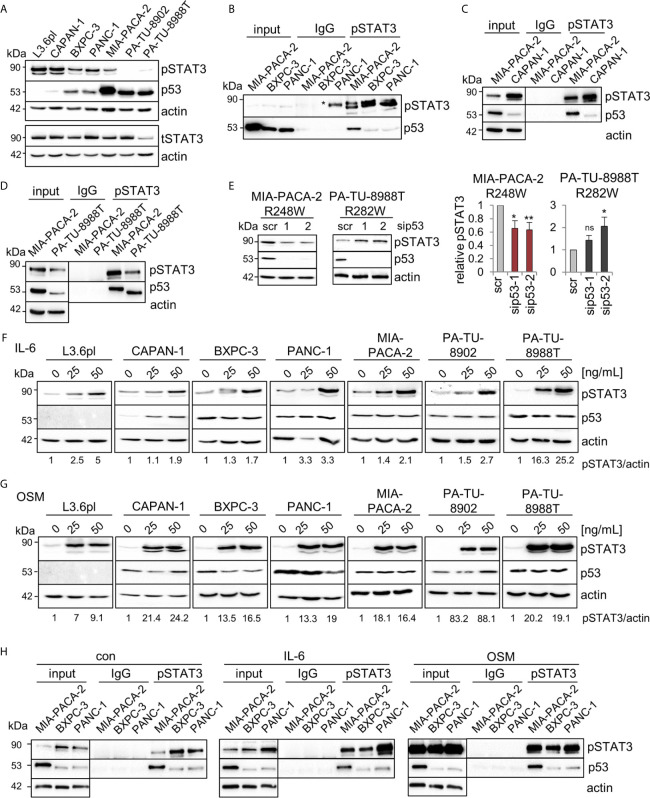
Mutp53^R248W^ selectively binds to phosphorylated STAT3 in PDAC cells. **(A)** Representative immunoblot analysis of seven different PDAC cell lines. pSTAT3, pTyr 705-STAT3 (Y705), and total STAT3 (tSTAT3). Actin, loading control. **(B–D)** Co-immunoprecipitations (CoIPs) of untreated MIA-PACA-2, PANC-1, BXPC-3 **(B)**, CAPAN-1 **(C)**, and PA-TU-8988T **(D)** cells using anti-pSTAT3 (Y705) or IgG antibodies followed by immunoblot analysis. MIA-PACA-2 cells were always used as positive control. Note that the pSTAT3 band marked by an asterisk in **(B)** is an artefact due to a leaky pocket from the neighboring MIA-PACA-2 lane. **(E)** Knockdown of mutp53 in MIA-PACA-2, but not in PA-TU-8988T cells downregulates pSTAT3 levels. Cells were transfected with two different siRNAs against *TP53* mRNA (sip53-1, -2) or scrambled control (scr) for 72 h followed by immunoblot analysis. Representative immunoblot out of 3 (MIA-PACA-2) and out of 4 (PA-TU-8988T). Actin, loading control. (right) Diagrams represent the means ± SEM of densitometric quantifications of three (MIA-PACA-2, n=3) or two (PA-TU-8988T, n = 4) independent experiments, normalized to actin. Calculated relative to control scrambled siRNA (scr). Student’s t test. *p ≤ 0.05; **p ≤ 0.01; ns, not significant. **(F, G)** Treatment of PDAC cell lines with the indicated concentrations of Interleukin-6 (IL-6, **F**), Oncostatin M (OSM, **G**), or respective solvent controls for 24 h. Representative immunoblot for pSTAT3 (Y705) induction is shown. Quantification by densitometry, normalized to actin loading control (pSTAT3/actin ratio) and calculated relative to solvent control. ‘pSTAT3/actin’, densitometric quantifications of the representative immunoblot, normalized to actin and relative to 0 ng/ml IL-6 or OSM treatments. **(H)** CoIPs of MIA-PACA-2, PANC-1 and BXPC-3 cells stimulated with 50 ng/mL IL-6, OSM or solvent control for 24 h. Immunoprecipitation using anti-pSTAT3 (Y705) or IgG antibodies, followed by immunoblot as indicated. Actin in unprecipitated input lysates, loading control. **(B–D, H)** Five percent of input were used for input control.

In colorectal carcinoma, an important mechanism of tumor invasion is mediated by mutp53^R248Q/W^-pSTAT3 signaling by forming a physical complex (26). Reduced migration capacity of MIA-PACA-2 cells after mutp53^R248W^ depletion (**Figures 3A, F**) suggests a similar mechanism. Since the STAT3 pathway is also an important driver of PDAC tumorigenesis (48, 49), we asked whether mutp53^R248W^-regulated migration is similarly mediated through STAT3 signaling. The PDAC panel showed high constitutive levels of phosphorylated STAT3 (pSTAT3) in five of the seven cell lines (**Figure 4A**). Only two cell lines, PA-TU-8902 and PA-TU-8988T, had very low levels of activated STAT3 (yet exhibited significant stabilization of mutp53). On the other hand, this immunoblot analysis that examines relative ratios of both proteins indicated that four lines with high pSTAT3 had very low or undetectable mutp53 levels. Importantly, MIA-PACA-2 cells, as the only cell line with dually high levels of both mutp53 and pSTAT3, seem to fulfill the best precondition to promote migration *via* this axis.

Thus, co-immunoprecipitations (CoIPs) were performed to test which of the various mutp53 proteins are able to bind STAT3. Indeed, R248W in MIA-PACA-2 cells showed the strongest binding to total STAT3 protein compared to BXPC-3 and PANC-1 cells, forming a constitutive endogenous signaling complex (**Supplementary Figure 2A**). Since phosphorylation status is another important parameter for binding to STAT3, these cell lines with different mutp53 variants and stabilization levels were subjected to CoIPs with an antibody specific for phosphorylated STAT3. Among these mutants analyzed, mutp53^R248W^ in MIA-PACA-2 cells again showed the strongest binding to pSTAT3 (**Figure 4B**). CAPAN-1 cells with low mutp53 level showed a minor binding to pSTAT3 (**Figure 4C**) such as BXPC-3 and PANC-1 cells (**Figure 4B**) (yet exhibited moderate levels of mutp53 compared to CAPAN-1). However, PA-TU-8988T cells with intermediate mutp53 levels (lower than in MIA-PACA-2 but higher than in PANC-1 or BXPC-3 cells) again showed a strong binding of mutp53^R282W^ to pSTAT3 (**Figure 4D**). This confirms a point made in colorectal carcinoma that the ability of mutp53 to bind to pSTAT3 correlates with the degree of its stabilization (26).

To investigate if the mutp53-pSTAT3 complex can directly regulate the phosphorylation status of STAT3 as shown in CRC (26), we depleted mutp53 in MIA-PACA-2, PA-TU-8988T, PANC-1, BXPC-3 and PA-TU-8902 cells (**Figure 4E** and **Supplementary Figure 2B**). In MIA-PACA-2 and PA-TU-8988T cells, both with a strong mutp53-pSTAT3 complex formation, only mutp53^R248W^ regulated STAT3 activity in PDAC cells, as indicated by decreased STAT3 phosphorylation selectively in MIA-PACA-2 cells (**Figure 4E**). In all other cell lines tested, pSTAT3 level were not decreased after mutp53 depletion (**Figure 4E** and **Supplementary Figure 2B**). Why mutp53 binding to pSTAT3 failed to reduce STAT3 activity in PA-TU-8988T cells remains speculative but confirms the reduced migration capacity after mutp53 depletion exclusively in MIA-PACA-2 cells (**Figures 3A–D**). These data further underline the strong invasive GOF function of the mutp53^R248W^ allele reaching across cancer entities.

Although most PDAC cell lines already exhibited high constitutive levels of pSTAT3 at baseline (**Figure 4A**), treatment with Interleukin-6 (**Figure 4F**) or Oncostatin M (**Figure 4G**) further stimulated the STAT3 pathway and induced additional increase in phosphorylated STAT3. Thus, to further evaluate whether the mutp53 binding capacity to pSTAT3 increases with higher pSTAT3 levels, MIA-PACA-2, as well as PANC-1 and BXPC-3 cells (both with a low binding capacity), were treated with IL-6, OSM, or solvent control. Interestingly, even after this strong induction of pSTAT3, the p53^R248W^ mutant showed by far the strongest binding to pSTAT3, again emphasizing allele selectivity (**Figure 4H**). These data suggest that it is not the level of pSTAT3 that predicts p53 binding in PDAC, but rather the nature of the mutp53 variant. In sum, mutp53^R248W^ shows a strong ability for complexing with pSTAT3 and regulation of migration, independent of the levels of phosphorylated STAT3.

### Mutp53^R248W^ Selectively Regulates STAT3 Phosphorylation and Activity in PDAC Cells

**Figure 5:**
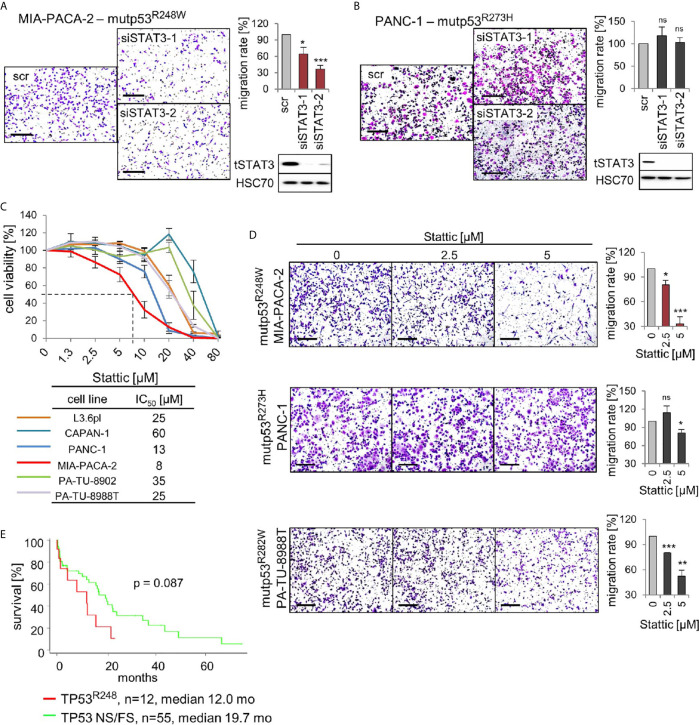
p53^R248W^ mutant selectively regulates STAT3 phosphorylation and activity in PDAC cells. **(A, B)** STAT3 knockdown phenocopies mutp53 knockdown in migration assays. MIA-PACA-2 **(A)** and PANC-1 **(B)** cells were transfected with two different siRNAs against *STAT3* mRNA (siSTAT3-1, -2) or scrambled control (scr). Seventy-two-hour post-transfection cells were seeded into transwell inserts to assess their migration. After 24 h, cells were fixed, stained, and counted at the membrane underside. Scale bars, 200 µm. MIA-PACA-2 cells: 4 biological replicates (n = 4), PANC-1 cells: 3 biological replicates, 2 out of 3 with 2 technical replicates (n = 5). Cells were calculated relative to scrambled control. Immunoblot analysis to confirm knockdown of STAT3. HSC70, loading control. **(C)** Cell viability assays of the indicated PDAC cell lines. Dose response curve after treatment with increasing concentrations of the STAT3 inhibitor Stattic or solvent control for 24 h. For each cell line, three to four biological replicates were measured. Diagram represents means ± SEM. From these curves, IC_50_ values were determined, indicated in the table. Of note, MIA-PACA-2 cells are the most sensitive to Stattic treatment, indicated by the dashed line. **(D)** STAT3 inhibition phenocopies mutp53 knockdown in migration assays. Transwell migration assays of MIA-PACA-2, PANC-1, and PA-TU-8988T cells treated with the indicated concentrations of Stattic for 24 h. Scale bars, 200 µm. For all cell lines, quantification of two biological replicates, one of them with two technical replicates (n = 3 total), calculated relative to 0 µM control treatment. **(E)** Survival curve of PDAC patients harboring TP53 R248 mutations versus patients harboring TP53 nonsense or frameshift (NS/FS) mutations. Number of patients and mean overall survival in months as indicated. TCGA data. Kaplan–Meier statistic, log-rank test. **(A, B, D)** Diagrams represent the means ± SEM. Student’s t test. *p ≤ 0.05; **p ≤ 0.01; ***p ≤ 0.001; ns, not significant.

The above findings led us to hypothesize that mutp53^R248W^ binds to and deregulates pSTAT3 in PDAC cells by forming an oncogenic complex. Since mutp53^R248W^ depletion also selectively suppressed phosphorylation and thus activation of STAT3 (**Figure 4E**), we next asked whether the R248W mutant can be functionally linked to STAT3 dependency for migration in PDAC cells. To this end, we determined migration capacity after STAT3 ablation. Indeed, depletion of STAT3 suppressed migration ability in mutp53^R248W^ expressing MIA-PACA-2 cells (**Figure 5A**) but not in mutp53^R273H^ expressing PANC-1 cells (**Figure 5B**).

To confirm that phosphorylated STAT3 is critical for the oncogenic mechanism of the tumor-promoting mutp53^R248W^-pSTAT3 complex, we used the small-molecule STAT3 inhibitor Stattic. Stattic selectively inhibits activation of STAT3 through interference with dimerization and nuclear translocation (50). It has been shown that Stattic substantially reduces STAT3 phosphorylation in colorectal, liver, and breast cancer cells (50–52) as well as in PDAC cells such as MIA-PACA-2 and PANC-1 (53–55). Importantly, among the panel of PDAC cells, R248W expressing MIA-PACA-2 cells were again the most susceptible to pSTAT3 inhibition by Stattic with the lowest IC_50_ value (8 µM) in cell viability assays (**Figure 5C**). Likewise, migration after Stattic treatment was strongly suppressed in MIA-PACA-2 cells (by 70%), but lower suppressed in PANC-1 (by 15%) or PA-TU-8988T cells (by 45%) cells (**Figure 5D**).

The mutp53^R248W^-pSTAT3 complex might accelerate tumor progression in PDAC patients as we had previously seen in CRC patients (26). Indeed, patient data support this notion since PDAC patients harboring *TP53^R248Q^* or *TP53^R248W^* mutations showed a trend for reduced survival compared to patients with loss-of-function NS+FS mutation (**Figure 5E**), supporting the mutp53^R248W^-pSTAT3 complex as a potentially attractive target in PDAC. Furthermore, we analyzed other missense mutants such as mutp53^R159^, mutp53^R175^, mutp53^Y220^, mutp53^R273^, and mutp53^R282^. However, TCGA data do not provide enough PDAC cases for a sufficient statistical analysis (**Supplementary Figure 3A**). Albeit we see a tendency that other stabilized missense p53 mutants shorten patient survival, which indeed might provide attractive targets as well, more analysis is needed to explore GOF activities that are acquired by other p53 mutants (**Supplementary Figure 3B**).

In conclusion, targeting the tumor-promoting mutp53^R248W^-pSTAT3 complex by STAT3 depletion or pharmacological inhibition diminished cell viability and migration in mutp53^R248W^ expressing, but not in mutp53^R273H^ expressing, PDAC cells.

## Discussion

The phenotype of p53 missense mutants is heterogenous and moreover depends on the cellular context (16–18). Here, we analyze a panel of p53 missense mutants (mutp53) in a series of homozygous human PDAC cell lines and compare the impact of various mutants on protein properties and functions. We find that mutp53^R248W^ protein undergoes strong Hsp90-mediated stabilization and selectively promotes migration by engaging in the strong constitutive complex formation with phosphorylated STAT3 at baseline and upon cytokine stimulation. Our data in pancreatic cancer suggest a R248W allele-specific gain-of-function on migration *via* STAT3 deregulation. These data mirror our previous findings in colorectal cancer (26) and further underline the necessity to investigate p53 missense mutants in a context- and allele-dependent manner (16, 19, 20).

Interestingly, PA-TU-8902 cells expressing intermediate stabilized mutp53^C176S^ showed strong STAT3 pathway stimulation by OSM or IL-6 (**Figures 4F, G**) but did not migrate at all in the transwell assay (**Figure 3E**), indicating that STAT3 fails to impact migration in these cells. Furthermore, PA-TU-8988T cells harboring intermediate levels of mutp53^R282W^ showed a strong binding to pSTAT3 but failed to regulate pSTAT3 level (indicating STAT3 activity) (**Figure 4E**) and failed to influence the migratory capacity in transwell assays as seen in mutp53^R248W^-containing MIA-PACA-2 cells (compare **Figures 3A, C**). However, in principle, the mutp53^R282W^-pSTAT3 complex confirms a point made in our colorectal carcinoma study that the ability of mutp53 to bind pSTAT3 correlates with the degree of its stabilization (26). The function that is acquired by the mutp53^R282W^-pSTAT3 complex in PA-TU-8988T remains speculative. STAT3 is not just an important factor for PDAC migration (54, 56, 57) but is also involved in many other hallmarks of cancer to promote tumor progression (58, 59).

Thus, we find that different p53 mutants have different impacts on migration- and cell growth-associated STAT3 functions. Importantly, among *TP53* mutations, several other common alterations exist that drive PDAC (41). We cannot exclude that molecular PDAC subtypes influence mutp53 GOF activities. Other mutations and alteration might also contribute to migratory differences after depletion of mutp53 variants. To address this question, an isogenic cell panel with various *TP53* mutations is necessary. Since the maintenance of the *TP53* copy number is very crucial in relation to mutp53 protein stabilization, a CRISPR/Cas9-based isogenic cell panel might be most useful.

Mechanistically, the favored GOF hypothesis is that the nuclear presence of highly abundant stabilized mutp53 proteins, which have lost specific DNA binding capacity on their own, results in hijacking of (by binding to) other transcription factors and their specific cofactors, thereby building a complex network to divert and oncogenically reprogram their transcriptional activity (5, 6, 20, 24, 60–62). Regarding co-factors, it is conceivable that the mutp53 protein also adds p53-specific coactivators into this illegitimate mix, and/or that the canonical coactivator specific for the partnering transcription factor might get displaced. Thus, interplay networks of mutp53 with co-regulation of various tumor drivers is essential for GOF-mediated cancer progression (4, 6, 24, 60, 63). This concept could explain why the mutp53 status or the status of STAT3 phosphorylation alone is not yet a determinant for migration but depends on the specific missense mutation, resulting in specific mutp53-pSTAT3 complexes with mutp53 variant-specific transcriptional cofactors. In line with this, it is shown that mutp53^R273H^ and mutp53^R175H^ can regulate NF-κB activity in cancer cells (64, 65). Interestingly, NF-κB and STAT3 also physically interact and coregulate transcriptional pathways in cancer (66, 67). Together with our finding that mutp53^R273H^ does not significantly bind to pSTAT3 in PANC-1 cells (**Figure 4B**) and does not regulate their migration (**Figures 3B, G**), it further emphasizes the allele specificity of oncogenic mechanisms. Other studies also show context-dependent mutp53 specificities (6, 17). One example is mutp53^R175H^, which promotes aberrant self-renewal in leukemic cells through binding to FOXH1 as critical regulator of stem cell–associated genes (68). Furthermore, mutp53^R175H^ or mutp53^R273H/C^ form complexes with NF-Y and p300 proteins to override cellular failsafe programs, thus permitting tumor progression (69). Mutp53 promotes invasion, e.g., *via* constitutive activation of EGFR/integrin signaling (70) and by antagonizing TAp63 (71).

Mutp53 stabilization occurs *via* binding to Hsp90 (5, 23), which offers therapeutic approaches to target stabilized GOF mutp53 protein in cancer cells *via* Hsp90 inhibition. Thus, treatment with the Hsp90 inhibitors Ganetespib and Onalespib diminished mutp53 levels in most analyzed PDAC cells (**Figure 2A**). However, in BXPC-3 cells, both Hsp90 inhibitors failed to destabilize Hsp90 clients (also see *Functional Control AKT*). The reason why remains speculative but resistance mechanisms are known such as an UGT1A (UDP glucuronosyltransferase 1A) overexpression (72). Importantly, in cells with a strong stabilization of mutp53 (MIA-PACA-2, PA-TU-8902, and PA-TU-8988T, **Figure 1B**), inhibition of Hsp90 resulted in significant suppression of cell growth (**Figure 2B**). In CAPAN-1 cells with a low degree of mutp53 stabilization (**Figures 1A, B**), Hsp90 inhibition did not substantially impact cell confluency (**Figure 2B**).

In sum, our preliminary *in vitro* results support a GOF of mutp53^R248W^ in pancreatic cancer, justifying future *in vivo* investigations on stabilized mutp53 as a putative therapeutic target in this important tumor entity that is in dire need of new therapeutic concepts.

## Data Availability Statement

The original contributions presented in the study are included in the article/**Supplementary Material**. Further inquiries can be directed to the corresponding author.

## Author Contributions

Conceptualization: RS-H, LK. Methodology: RS-H, LK, CF, SS. Experimentation: LK, CF, NW, FT. Writing Original Draft LK, RS-H, UM. Writing Review and Editing: all authors. Funding Acquisition: RS-H, UM. Supervision: RS-H. All authors contributed to the article and approved the submitted version.

## Funding

RS-H and LK are supported by the Deutsche Forschungsgemeinschaft (DFG, SCHUH-3160/3-1). RS-H and SS are supported by the Clinical Research Unit KFO5002 (DFG, SCHUH-3160/4-1 and SI-2639/1-1). UM is supported by the NIH National Cancer Institute (2R01CA176647).

## Conflict of Interest

The authors declare that the research was conducted in the absence of any commercial or financial relationships that could be construed as a potential conflict of interest.
