# Supplementary material for: The Gain-of-Function p53 R248W Mutant Promotes Migration by STAT3 Deregulation in Human Pancreatic Cancer Cells
Source: Front Oncol. 2021 Jun 11;11:642603. doi: 10.3389/fonc.2021.642603 (PMC8226097; doi:10.3389/fonc.2021.642603)
Supplement: Supplementary file 1 [file DataSheet_1.pdf]

Index	Gene	Transcript	Locatior	Pos.	Type	Nuc Change	Coverage	Char	C	H	web Ref.	c. HGVS	p. I	mu	m	TVa	MV	Weightin	Filter Step
1	GNAS	ENST0000037108	E1	-37411 [c	C	G -> A (het)	22% (356) [22% (171) / 22% (185)]				rs74934823 (10	c.-37835G>A							distinct
2	MAPK4	ENST0000040038	E3	-10553 [c	C	C -> T (homo)	100% (705) [100% (442) / 100% (263)]				rs7227230 (100	c.547-10553C>T							distinct
3	FAT4	ENST0000039432	E2	-4905 [ch	C	T -> C (homo)	100% (759) [100% (524) / 100% (235)]				rs925243 (1000	c.5176-4905T>C							distinct
4	BRD2	ENST0000044908	E1	-3818 [ch	C	G -> A (homo)	99% (427) [100% (348) / 96% (79)]				rs116656352 (1	c.-3976G>A							distinct
5	BRD2	ENST0000044908	E1	-3813 [ch	C	C -> A (homo)	99% (466) [100% (379) / 97% (87)]				rs115634162 (1	c.-3971C>A							distinct
6	LMNA	ENST0000036830	E2	-778 [chr	C	C -> G (homo)	100% (291) [99% (159) / 100% (132)]				rs665979 (1000	c.357-778C>G							distinct
7	LMNA	ENST0000036830	E2	-739 [chr	C	T -> G (homo)	100% (306) [100% (137) / 100% (169)]				rs513043 (1000	c.357-739T>G		0/2 of 4					distinct
8	BRD2	ENST0000044908	E7	-232 [chr	C	A -> G (homo)	99% (391) [100% (235) / 99% (156)]				rs3129307 (100	c.1060-232A>G							distinct
9	BRD2	ENST0000044908	E9	-53..-51 /	D	CTT (homo)	93% (589) [95% (333) / 91% (256)]					c.1438-53_1438-51delCTT							distinct
10	KMT2D	ENST0000030106	E32	-46 [chr1	C	G -> A (het)	33% (378) [32% (202) / 33% (176)]					c.8047-46G>A							distinct
11	LMNA	ENST0000036830	E7	-43 [chr1	C	A -> G (het)	64% (656) [68% (273) / 62% (383)]				rs16837198 (10	c.1158-43A>G							distinct
12	SLIT2	ENST0000050415	E28	-42 [chr4:	C	A -> G (het)	31% (267) [28% (104) / 33% (163)]				rs2290750 (100	c.2851-42A>G							distinct
13	SMARCA4	ENST0000045071	E10	-37..-36 /	Indel	TG -> CA (homo)	92% (898) [94% (421) / 91% (477)]				rs386806808 (d	762-37_1762-36delinsCA							distinct
14	MYCBP2	ENST0000035733	E42	-20 [chr1	C	A -> G (homo)	100% (320) [100% (233) / 100% (87)]				rs3742103 (100	c.6001-20A>G							distinct
15	SMARCA2	ENST0000034972	E9	-19 [chr9:	C	G -> A (het)	36% (199) [33% (103) / 39% (96)]				rs141618610 (1	c.1522-19G>A							distinct
16	LRP1B	ENST0000038948	E36	-17 / 1bp	I (Dup)	T (het)	51% (207) [54% (168) / 43% (39)]				rs398104769 (d	c.5759-18dupT							distinct
17	FGFR2	ENST0000035848	E14	-17 [chr1	C	T -> G (het)	46% (203) [48% (143) / 44% (60)]				rs3135802 (100	c.1864-17T>G							distinct
18	LRP1B	ENST0000038948	E34	-17 [chr2:	C	A -> G (homo)	100% (545) [100% (323) / 100% (222)]				rs7599219 (dbS	c.5501-17A>G							distinct
19	TLE4	ENST0000037655	E11	-16 [chr9:	C	C -> T (homo)	100% (235) [100% (206) / 100% (29)]				rs73652238 (10	c.784-16C>T							distinct
20	MARK2	ENST0000040201	E18	-16 [chr1	C	C -> T (het)	25% (258) [24% (132) / 25% (126)]					c.1962-16C>T							distinct
21	KMT2C	ENST0000026218	E18	-15 [chr7:	C	T -> C (het)	43% (178) [46% (120) / 39% (58)]				rs62481502 (db	c.2872-15T>C							distinct
22	SF3B1	ENST0000033550	E19	-15 [chr2:	C	A -> T (homo)	100% (209) [100% (169) / 100% (40)]				rs788017 (1000	c.2719-15A>T							distinct
23	LRP1B	ENST0000038948	E45	-15 [chr2:	C	C -> G (het)	57% (218) [61% (142) / 52% (76)]				rs11694934 (10	c.7388-15C>G							distinct
24	ROBO3	ENST0000039780	E3	-14 [chr1	C	A -> C (het)	69% (11) [100% (11) / 0% (0)]				rs11219820 (10	c.488-14A>C							forced,distinct
25	EGFR	ENST0000027549	E1	-14 [chr7:	C	A -> C (homo)	100% (85) [100% (80) / 100% (5)]				rs712830 (1000	c.-191A>C							forced,distinct
26	BRD2	ENST0000044908	E2	-13 / 1bp	I (Dup)	T (homo)	93% (91) [93% (86) / 100% (5)]					c.-112-14dupT							forced,distinct
27	SLIT2	ENST0000050415	E12	-12 [chr4:	C	T -> C (homo)	100% (235) [100% (154) / 100% (81)]				rs519813 (1000	c.1059-12T>C							distinct
28	SF3B1	ENST0000033550	E13	-11 / 2bp	I (Dup)	TT (homo)	86% (166) [86% (129) / 86% (37)]				rs397804827 (d	1720-13_1720-12dupTT							distinct
29	ROBO2	ENST0000048769	E2	-10 [chr3:	C	C -> G (het)	79% (1972) [78% (1215) / 80% (757)]				rs9631539 (dbS	c.-13-10C>G							distinct
30	BRD2	ENST0000044908	E3	-10 [chr6:	C	T -> C (homo)	99% (256) [99% (193) / 100% (63)]				rs635688 (1000	c.193-10T>C							distinct
31	MYCBP2	ENST0000035733	E13	-10 / 1bp	I (Dup)	T (het)	55% (126) [69% (101) / 30% (25)]				rs549452634 (d	c.1534-11dupT							distinct
32	KRAS	ENST0000025607	E6	-9 [chr12:	C	G -> A (het)	37% (117) [38% (78) / 34% (39)]				rs12313763 (10	c.*5-9G>A							distinct
33	SLIT2	ENST0000050383	E9	-9 [chr4:g	C	G -> T (het)	30% (191) [30% (105) / 30% (86)]				rs7695303 (100	c.776-9G>T							distinct
34	KMT2D	ENST0000030106	E11	-7 / 1bp [	D	C (homo)	96% (946) [96% (401) / 96% (545)]				rs112620957 (d	c.2798-7delC							distinct
35	BRAF	ENST0000028860	E1	-7 [chr7:g	C	A -> C (het)	29% (7) [32% (7) / 0% (0)]					c.-68A>C							forced,distinct
36	LRP1B	ENST0000038948	E27	-7 [chr2:g	C	T -> G (het)	51% (81) [53% (70) / 44% (11)]				rs12987572 (10	c.4335-7T>G							distinct
37	NARF	ENST0000030979	E9	-6 [chr17:	C	T -> G (het)	74% (704) [73% (336) / 76% (368)]				rs12103494 (10	c.834-6T>G							distinct
38	ROBO1	ENST0000046423	E22	-6 / 1bp [	I (Dup)	T (het)	83% (179) [88% (127) / 74% (52)]				rs369027577 (d	c.2883-7dupT							distinct
39	APC	ENST0000025743	E5	-4 / 1bp [	D	A (het)	74% (111) [82% (58) / 68% (53)]				COSM19338 (C	c.423-4delA							distinct
40	BRD2	ENST0000044908	E8	-4 [chr6:g	C	G -> C (homo)	100% (981) [100% (597) / 100% (384)]				rs3097644 (100	c.1189-4G>C							distinct
41	FAT1	ENST0000044180	E3	-4 [chr4:g	C	G -> C (homo)	100% (354) [100% (259) / 100% (95)]				rs172903 (1000	c.3266-4G>C							distinct
42	PBRM1	ENST0000039483	E11	-4 [chr3:g	C	G -> A (het)	74% (287) [65% (104) / 81% (183)]				COSM4002750	c.996-4G>A							distinct
43	LRP1B	ENST0000038948	E9	3 (1239) [	C	T -> C (het)	65% (77) [67% (74) / 38% (3)]	V (413			rs1525579 (100	c.1239T>C	p.Val413=						forced,distinct
44	LRP1B	ENST0000038948	E50	5 (8031) [	C	A -> G (het)	32% (122) [31% (73) / 33% (49)]	Q (267			COSM4001201	c.8031A>G	p.Gln2677=						distinct
45	CDKN2A	ENST0000030449	E2	5 (155) [c	C	T -> A (homo)	100% (932) [100% (567) / 100% (365)]	> K (52			COSM13436 (C	c.155T>A	p.Met52Lys						distinct

46	LRP1B	ENST0000038948	E54	5 (8526) [	C	T -> C (het)	30% (123)	[30% (78) / 30% (45)]	Y (2842	COSM3757652	c.8526T>C	p.Tyr2842=	distinct
47	ROBO3	ENST0000039780	E21	5 (2991) [	C	G -> A (homo)	100% (569)	[100% (327) / 100% (242)]	A (997	rs7933204 (100	c.2991G>A	p.Ala997=	distinct
48	MYCBP2	ENST0000035733	E55	8 (7778) [	C	A -> G (homo)	100% (542)	[100% (316) / 100% (226)]	S (2593	rs34474844 (10	c.7778A>G	p.Asn2593Ser	distinct
49	EGFR	ENST0000027549	E23	8 (2709) [	C	T -> C (homo)	100% (1254)	[100% (646) / 100% (608)]	T (903	rs1140475 (100	c.2709T>C	p.Thr903=	distinct
50	SF3B1	ENST0000033550	E5	8 (423) [c	C	A -> G (homo)	100% (380)	[100% (163) / 100% (217)]	K (141	COSM1129389	c.423A>G	p.Lys141=	distinct
51	POLR3A	ENST0000037237	E23	9 (2997) [	C	G -> T (het)	55% (377)	[59% (190) / 52% (187)]	V (999	rs12241228 (10	c.2997G>T	p.Val999=	distinct
52	JAG1	ENST0000025495	E20	10 (2382)	C	C -> T (het)	62% (318)	[64% (223) / 60% (95)]	S (794	rs56225585 (10	c.2382C>T	p.Ser794=	distinct
53	FAT4	ENST0000039432	E10	20 (11814)	C	A -> G (het)	68% (262)	[70% (180) / 65% (82)]	S (3938	rs17009721 (10	c.11814A>G	p.Ser3938=	distinct
54	KMT2C	ENST0000026218	E7	22 (871) [	C	C -> T (het)	78% (538)	[81% (387) / 71% (151)]	F (291	rs56850341 (10	c.871C>T	p.Leu291Phe	distinct
55	POLR3A	ENST0000037237	E16	26 (2100)	C	C -> T (het)	67% (629)	[67% (346) / 67% (283)]	I (700)	rs79793998 (10	c.2100C>T	p.Ile700=	distinct
56	FAT1	ENST0000044180	E11	26 (8904)	C	C -> T (homo)	100% (277)	[100% (226) / 100% (51)]	A (2968	rs1280099 (100	c.8904C>T	p.Ala2968=	distinct
57	SLC25A24	ENST0000056548	E1	27 [chr1:g	C	T -> G (het)	78% (101)	[78% (100) / 100% (1)]	' UTR	rs662282 (1000	c.-194T>G		forced,distinct
58	U2AF1	ENST0000029155	E1	29 [chr21	C	A -> C (het)	49% (298)	[48% (166) / 49% (132)]	' UTR	rs17115876 (10	c.-65A>C		distinct
59	MYCBP2	ENST0000035733	E62	29 (10440)	C	G -> A (homo)	100% (236)	[100% (139) / 100% (97)]	P (3480	rs34700794 (10	c.10440G>A	p.Pro3480=	distinct
60	FAT4	ENST0000039432	E16	30 (12846)	C	C -> T (het)	71% (280)	[73% (187) / 67% (93)]	S (4282	rs17009819 (10	c.12846C>T	p.Ser4282=	distinct
61	ROBO2	ENST0000048769	E2	32 (19) [c	C	C -> A (het)	79% (1720)	[78% (832) / 80% (888)]	-> S (7)	rs12171318 (db	c.19C>A	p.Arg7Ser	distinct
62	NARF	ENST0000030979	E9	34 (867) [	C	T -> C (het)	78% (726)	[76% (307) / 80% (419)]	R (289	rs3829567 (100	c.867T>C	p.Arg289=	distinct
63	MYCBP2	ENST0000035733	E46	37 (6520)	C	C -> T (homo)	100% (329)	[100% (220) / 100% (109)]	L (2174	rs34982494 (10	c.6520C>T	p.Leu2174=	distinct
64	FGFR2	ENST0000035848	E1	38 [chr10	C	G -> A (homo)	100% (275)	[100% (80) / 100% (195)]	' UTR	rs1047111 (100	c.-236G>A		distinct
65	SMARCC2	ENST0000055016	E5	39 (438) [	C	A -> G (homo)	99% (785)	[99% (318) / 100% (467)]	P (146	rs7136420 (100	c.438A>G	p.Pro146=	distinct
66	BANF1	ENST0000031217	E1	39 / 2bp [	I (Dup)	AG (het)	42% (291)	[41% (165) / 44% (126)]	' UTR		c.-472_-471dupAG		distinct
67	PIK3CA	ENST0000026396	E12	42 (1788)	C	A -> G (het)	62% (572)	[65% (322) / 59% (250)]	E (596	rs137902538 (1	c.1788A>G	p.Glu596=	distinct
68	TLE4	ENST0000037655	E18	42 (2028)	C	A -> C (homo)	100% (959)	[100% (447) / 100% (512)]	A (676	rs34566811 (10	c.2028A>C	p.Ala676=	distinct
69	KRAS	ENST0000025607	E2	46 (35) [c	C	G -> A (het)	48% (318)	[48% (150) / 48% (168)]	> D (12)	rs121913529 (C	c.35G>A	p.Gly12Asp	distinct
70	LRP1B	ENST0000038948	E32	47 (5256)	C	A -> G (homo)	100% (721)	[100% (412) / 100% (309)]	S (1752	COSM4133144	c.5256A>G	p.Ser1752=	distinct
71	ARID2	ENST0000033434	E1	52 [chr12	C	G -> A (het)	46% (6)	[50% (6) / 0% (0)]	' UTR		c.-121G>A		forced,distinct
72	SLC25A24	ENST0000037004	E1	53 [chr1:g	C	A -> T (homo)	100% (91)	[100% (91) / 0% (0)]	' UTR	rs524504 (1000	c.-145A>T		forced,distinct
73	ROBO2	ENST0000048769	E2	55 (42) [c	C	A -> G (het)	79% (2134)	[76% (893) / 82% (1241)]	> T (14)	rs62269817 (db	c.42A>G	p.Thr14=	distinct
74	SMARCA4	ENST0000045071	E5	56 (915) [	C	G -> A (het)	95% (485)	[47% (21) / 100% (464)]	P (305	rs149573400 (1	c.915G>A	p.Pro305=	distinct
75	LRP1B	ENST0000038948	E27	58 (4392)	C	A -> G (het)	43% (180)	[44% (118) / 41% (62)]	R (1464	rs79054985 (10	c.4392A>G	p.Arg1464=	distinct
76	KMT2C	ENST0000026218	E15	59 (2591)	C	A -> G (het)	35% (69)	[41% (56) / 22% (13)]	G (864	rs4024420 (dbS	c.2591A>G	p.Glu864Gly	distinct
77	POLR3A	ENST0000037237	E3	60 (240) [	C	A -> C (het)	74% (330)	[76% (206) / 71% (124)]	> L (80)	rs12248310 (10	c.240A>C	p.Leu80=	distinct
78	PBRM1	ENST0000039483	E23	64 (3522)	C	A -> T (homo)	100% (904)	[100% (389) / 100% (515)]	P (1174	COSM4002747	c.3522A>T	p.Pro1174=	distinct
79	BRD2	ENST0000044908	E2	64 [chr6:g	C	G -> A (homo)	100% (405)	[100% (286) / 100% (119)]	' UTR	rs516535 (1000	c.-49G>A		distinct
80	LRP1B	ENST0000038948	E30	67 (5006)	C	A -> C (het)	65% (639)	[67% (399) / 63% (240)]	A (1669)		c.5006A>C	p.Glu1669Ala	distinct
81	SLIT2	ENST0000050415	E21	68 (2211)	C	C -> T (het)	28% (204)	[28% (107) / 28% (97)]	V (737	rs7690492 (100	c.2211C>T	p.Val737=	distinct
82	APC	ENST0000025743	E14	69 (1695)	C	A -> G (homo)	100% (241)	[100% (102) / 99% (139)]	E (565	rs77921116 (10	c.1695A>G	p.Glu565=	distinct
83	LRP1B	ENST0000038948	E8	69 (1082)	C	G -> A (het)	36% (279)	[35% (131) / 36% (148)]	Q (361	COSM1007086	c.1082G>A	p.Arg361Gln	distinct
84	SLC25A24	ENST0000056548	E1	70..76 / 7b	D	CCTGCGC (het)	65% (112)	[65% (110) / 50% (2)]	' UTR	rs150513527 (1	.-151_-145delCCTGCGC		forced,distinct
85	FAT1	ENST0000044180	E23	74 (12177)	C	G -> C (homo)	100% (641)	[100% (300) / 100% (341)]	N (4059	rs1280097 (100	c.12177G>C	p.Lys4059Asn	distinct
86	RBM6	ENST0000026602	E21	75 (3321)	C	C -> T (homo)	100% (371)	[100% (112) / 100% (259)]	Y (1107	COSM4158172	c.3321C>T	p.Tyr1107=	distinct
87	CHD1	ENST0000028404	E26	77 (3648)	C	C -> T (homo)	100% (670)	[100% (350) / 100% (320)]	S (1216	rs140751250 (1	c.3648C>T	p.Ser1216=	distinct
88	EGFR	ENST0000027549	E20	78 (2361)	C	G -> A (homo)	99% (821)	[98% (427) / 99% (394)]	Q (787	COSM1451600	c.2361G>A	p.Gln787=	distinct
89	LRP1B	ENST0000038948	E85	80 (13047)	C	G -> A (het)	22% (220)	[24% (108) / 21% (112)]	T (4349	rs1386356 (100	c.13047G>A	p.Thr4349=	distinct
90	GNAS	ENST0000037108	E5	81 (393) [	C	C -> T (het)	81% (740)	[82% (276) / 81% (464)]	I (131)	COSM3758661	c.393C>T	p.Ile131=	distinct

91	SLC25A24	ENST0000056548	E4	82 (480) [	C	G -> A (het)	28% (139)	[25% (62) / 31% (77)]	E (160	rs11185293 (10	c.480G>A	p.Glu160=	distinct
92	PIK3CA	ENST0000026396	E6	84 (1143)	C	C -> G (het)	63% (491)	[61% (178) / 64% (313)]	P (381	rs72561481 (10	c.1143C>G	p.Pro381=	distinct
93	ROBO2	ENST0000048769	E2	86..88 (73.	Indel	GTG -> ATC (het)	34% (891)	[32% (326) / 36% (565)]	> I (25)		c.73_75delinsATC	p.Val25Ile	distinct
94	KMT2C	ENST0000026218	E18	87 (2958)	C	A -> G (het)	51% (269)	[53% (123) / 50% (146)]	P (986	rs28439884 (db	c.2958A>G	p.Pro986=	distinct
95	CHD1	ENST0000028404	E31	87 (4335)	C	G -> A (homo)	100% (305)	[100% (112) / 100% (193)]	E (1445	COSM4003637	c.4335G>A	p.Glu1445=	distinct
96	ROBO2	ENST0000048769	E2	88 (75) [c	C	G -> C (het)	39% (977)	[36% (347) / 42% (630)]	> V (25)	rs62269818 (db	c.75G>C	p.Val25=	distinct
97	LRP1B	ENST0000038948	E51	89 (8238)	C	G -> A (het)	65% (284)	[65% (127) / 65% (157)]	G (274	rs61732738 (10	c.8238G>A	p.Gly2746=	distinct
98	SLC25A24	ENST0000056548	E1	89 [chr1:	C	A -> C (het)	75% (115)	[76% (113) / 50% (2)]	5' UTR	rs554709 (1000	c.-132A>C		forced,distinct
99	KMT2C	ENST0000026218	E18	92 (2963)	C	G -> T (het)	55% (256)	[56% (112) / 54% (144)]	F (988	rs28522267 (db	c.2963G>T	p.Cys988Phe	distinct
100	BRD2	ENST0000044908	E8	92 (1280)	C	C -> T (homo)	100% (807)	[100% (418) / 100% (389)]	V (427	rs3918143 (100	c.1280C>T	p.Ala427Val	distinct
101	SLC25A24	ENST0000056548	E2	93 (276) [	C	A -> G (het)	71% (195)	[70% (85) / 73% (110)]	> K (92)	COSM3750202	c.276A>G	p.Lys92=	distinct
102	BRD4	ENST0000026337	E11	95 (2142)	C	C -> T (homo)	100% (968)	[100% (444) / 100% (524)]	S (714	rs114723577 (1	c.2142C>T	p.Ser714=	distinct
103	KMT2C	ENST0000026218	E7	97 (946) [	C	A -> T (het)	83% (821)	[83% (469) / 82% (352)]	S (316	rs10454320 (db	c.946A>T	p.Thr316Ser	distinct
104	ATM	ENST0000027861	E23	99 (3383)	C	A -> G (homo)	100% (523)	[99% (136) / 100% (387)]	R (112	COSM1350830	c.3383A>G	p.Gln1128Arg	distinct
105	FAT1	ENST0000044180	E26	101 (1310	C	T -> C (homo)	100% (840)	[100% (404) / 100% (436)]	S (4367	COSM4158952	c.13101T>C	p.Ser4367=	distinct
106	SMARCA4	ENST0000045071	E8	105 (1524)	C	T -> C (homo)	100% (955)	[100% (480) / 100% (475)]	H (508	COSM4131184	c.1524T>C	p.H [c.1524T>C]	distinct
107	SMAD4	ENST0000034298	E9	112 (1067)	C	C -> T (homo)	100% (733)	[100% (368) / 100% (365)]	L (356	COSM14049 (C	c.1067C>T	p.Pro356Leu	distinct
108	FAT1	ENST0000044180	E19	112 (1066	C	T -> G (homo)	100% (779)	[100% (403) / 100% (376)]	A (355	rs2637777 (100	c.10660T>G	p.Ser3554Ala	distinct
109	LRP1B	ENST0000038948	E16	113 (2616)	C	C -> T (homo)	100% (160)	[100% (76) / 100% (84)]	D (872	rs13007735 (10	c.2616C>T	p.Asp872=	distinct
110	FGFR2	ENST0000035848	E1	117 [chr1	C	A -> G (het)	44% (65)	[44% (23) / 44% (42)]	5' UTR	rs41258305 (10	c.-157A>G		distinct
111	EGFR	ENST0000027549	E15	117 (1839)	C	C -> T (homo)	100% (676)	[100% (311) / 100% (365)]	A (613	rs17290169 (10	c.1839C>T	p.Ala613=	distinct
112	TLE4	ENST0000037655	E15	118 (1458)	C	C -> T (homo)	98% (1502)	[98% (751) / 98% (751)]	H (486	rs61742686 (10	c.1458C>T	p.His486=	distinct
113	SF3B1	ENST0000033550	E24	118 (3657)	C	A -> G (homo)	100% (721)	[100% (271) / 100% (450)]	V (121	COSM3757859	c.3657A>G	p.Val1219=	distinct
114	SLIT2	ENST0000050415	E1	120 [chr4	C	G -> T (het)	26% (10)	[26% (10) / 0% (0)]	5' UTR	rs7655084 (100	c.-133G>T		forced,distinct
115	TP53BP2	ENST0000034353	E6	121 (595)	C	G -> A (het)	60% (380)	[58% (165) / 61% (215)]	M (19	rs146703239 (1	c.595G>A	p.Val199Met	distinct
116	FAT1	ENST0000044180	E13	122 (9351)	C	T -> C (homo)	100% (1066)	[100% (477) / 100% (589)]	D (311	COSM4003024	c.9351T>C	p.Asp3117=	distinct
117	SMARCC2	ENST0000055016	E25	134 (2682)	C	C -> T (homo)	100% (291)	[100% (101) / 100% (190)]	A (894	rs17852368 (10	c.2682C>T	p.Ala894=	distinct
118	FAT1	ENST0000044180	E13	134 (9363)	C	C -> T (homo)	100% (1039)	[100% (413) / 100% (626)]	N (312	rs2249916 (100	c.9363C>T	p.Asn3121=	distinct
119	SF3B1	ENST0000033550	E18	135 (2631)	C	T -> C (homo)	100% (412)	[100% (128) / 100% (284)]	G (877	COSM3757860	c.2631T>C	p.Gly877=	distinct
120	BRD7	ENST0000039468	E7	144 (846)	C	C -> T (het)	50% (356)	[51% (163) / 50% (193)]	A (282	COSM3720984	c.846C>T	p.Ala282=	distinct
121	U2AF2	ENST0000045055	E11	159 (1191)	C	C -> T (homo)	99% (507)	[99% (193) / 99% (314)]	D (397	COSM1396639	c.1191C>T	p.Asp397=	distinct
122	TP53	ENST0000026930	E4	171 (267)	D	C (homo)	95% (635)	[91% (272) / 98% (363)]	122 R	COSM1180853	c.267delC	p.Ser90Profs*33	distinct
123	FAT1	ENST0000044180	E5	176 (3818)	C	A -> G (homo)	100% (991)	[100% (478) / 100% (513)]	R (127	rs328418 (1000	c.3818A>G	p.His1273Arg	distinct
124	FAT4	ENST0000039432	E4	191 (5760)	C	T -> C (homo)	100% (627)	[100% (295) / 100% (332)]	D (192	COSM1131273	c.5760T>C	p.Asp1920=	distinct
125	FGFR1	ENST0000044771	E1	192 [chr8	C	C -> T (homo)	100% (13)	[100% (10) / 100% (3)]	5' UTR	rs2445003 (100	c.-751C>T		distinct
126	FGFR2	ENST0000036905	E17	196 [chr1	C	A -> G (homo)	85% (23)	[0% (0) / 85% (23)]	3' UTR	rs1649167 (100	c.*190A>G		forced,distinct
127	LRP1B	ENST0000038948	E41	206 (6633)	C	A -> T (het)	32% (220)	[37% (102) / 29% (118)]	P (221	rs13431727 (10	c.6633A>T	p.Pro2211=	distinct
128	FAT1	ENST0000044180	E19	209 (1075	C	T -> C (het)	63% (545)	[63% (259) / 63% (286)]	T (358	rs115705222 (1	c.10757T>C	p.Met3586Thr	distinct
129	BRD3	ENST0000030340	E6	222 (936)	C	A -> G (homo)	100% (933)	[100% (440) / 100% (493)]	L (312	COSM4163507	c.936A>G	p.Leu312=	distinct
130	TGFBR2	ENST0000029575	E1	255 [chr3	C	C -> G (het)	76% (212)	[76% (192) / 83% (20)]	5' UTR	rs2306856 (100	c.-128C>G		distinct
131	FAT4	ENST0000039432	E9	257 (7701)	C	G -> C (homo)	100% (817)	[100% (399) / 100% (418)]	V (256	rs988863 (1000	c.7701G>C	p.Val2567=	distinct
132	BANF1	ENST0000031217	E1	260 [chr1	C	G -> C (het)	50% (449)	[51% (255) / 49% (194)]	5' UTR	rs1786171 (100	c.-249G>C		distinct
133	BANF1	ENST0000031217	E3	275 [chr1	C	C -> A (het)	52% (123)	[54% (19) / 52% (104)]	3' UTR	rs144367403 (1	c.*128C>A		distinct
134	PBRM1	ENST0000039483	E17	287 (2211)	C	A -> G (homo)	100% (879)	[100% (457) / 100% (422)]	T (737	COSM1566683	c.2211A>G	p.Thr737=	distinct

135	SLIT2	ENST0000050415	E37	293	[chr4	C	G -> A (homo)	100% (809)	[100% (303) / 100% (506)]	' UTR	rs1379659 (100	c.*51G>A		distinct
136	FGFR1	ENST0000044771	E1	307	[chr8	C	C -> T (het)	33% (10)	[35% (8) / 29% (2)]	' UTR	rs3213849 (100	c.-636C>T		forced,distinct
137	LMNA	ENST0000036830	E12	392	[chr1	C	C -> T (het)	75% (6)	[67% (2) / 80% (4)]	' UTR	rs74116489 (10	c.*365C>T		distinct
138	SETD2	ENST0000040979	E3	470 (557)		C	C -> T (homo)	100% (590)	[100% (268) / 100% (322)]	L (186	rs78759480 (10	c.557C>T	p.Pro186Leu	distinct
139	ACVR2A	ENST0000024141	E1	472 / 1bp		I (Dup)	T (het)	33% (4)	[33% (4) / 0% (0)]	' UTR		c.-166dupT		forced,distinct
140	BRD2	ENST0000037482	E2	523	[chr6	C	G -> T (homo)	100% (312)	[100% (57) / 100% (255)]	' UTR	rs974357 (1000	c.-782G>T		distinct
141	MARK2	ENST0000040201	E19	548	[chr1	C	A -> C (het)	47% (28)	[0% (0) / 47% (28)]	' UTR	rs182664 (1000	c.*187A>C		forced,distinct
142	RNF43	ENST0000040797	E9	633 (1585)		C	C -> T (het)	75% (1042)	[76% (639) / 74% (403)]	W (52	COSM191575 (	c.1585C>T	p.Arg529Trp	distinct
143	MYC	ENST0000037797	E2	708 (738)		C	G -> A (het)	55% (945)	[50% (303) / 58% (642)]	P (246	rs2070582 (100	c.738G>A	p.Pro246=	distinct
144	MYC	ENST0000037797	E2	708 (738)		C	G -> A (het)	55% (945)	[50% (303) / 58% (642)]	P (246	rs2070582 (100	c.738G>A	p.Pro246=	distinct
145	FAT1	ENST0000044180	E27	883	[chr4	C	G -> A (het)	30% (9)	[0% (0) / 30% (9)]	' UTR	rs7680937 (100	c.*254G>A		forced,distinct
146	LRP1B	ENST0000038948	E1	895	[chr2	C	C -> G (homo)	99% (705)	[99% (453) / 99% (252)]	' UTR	rs1375610 (100	c.-78C>G		distinct
147	KMT2D	ENST0000030106	E10	1025..105		D	ATCTCCGCAGGCT	74% (366)	[55% (98) / 85% (268)]	L -> L (	rs375538882 (dlATCTCCGCAGGCTGA		p.Ala765_Gln77	distinct
148	KMT2D	ENST0000030106	E10	1025..105		D	ATCTCCGCAGGCT	74% (366)	[55% (98) / 85% (268)]	L -> L (	rs375538882 (dlATCTCCGCAGGCTGA		p.Ala765_Gln77	distinct
149	TLE4	ENST0000037655	E1	1063 (45)		C	A -> G (homo)	100% (751)	[100% (291) / 100% (460)]	> P (15)	COSM4164047	c.45A>G	p.Pro15=	distinct
150	FAT1	ENST0000044180	E2	1230 (121		C	T -> G (het)	28% (257)	[30% (160) / 26% (97)]	R (404	COSM4003044	c.1212T>G	p.Ser404Arg	distinct
151	FAT4	ENST0000039432	E1	1371 (135		C	A -> T (homo)	100% (1316)	[100% (665) / 100% (651)]	> L (453	COSM3760369	c.1358A>T	p.Gln453Leu	distinct
152	FAT4	ENST0000039432	E9	1392 (883		C	A -> G (het)	59% (484)	[59% (245) / 59% (239)]	V (294	rs76048257 (10	c.8836A>G	p.Ile2946Val	distinct
153	FAT1	ENST0000044180	E2	1462 (144		C	G -> A (het)	29% (308)	[27% (134) / 31% (174)]	> I (482	rs3733413 (100	c.1444G>A	p.Val482Ile	distinct
154	FAT1	ENST0000044180	E2	1503 (148		C	C -> T (het)	28% (334)	[27% (174) / 30% (160)]	N (495	COSM4003040	c.1485C>T	p.Asn495=	distinct
155	FAT4	ENST0000039432	E17	1520 (145		C	A -> G (het)	64% (560)	[67% (271) / 61% (289)]	R (486	rs1014866 (100	c.14598A>G	p.Arg4866=	distinct
156	APC	ENST0000025743	E16	1774 (373		C	A -> G (homo)	100% (578)	[100% (242) / 100% (336)]	Q (124	rs74380081 (10	c.3732A>G	p.Gln1244=	distinct
157	FAT4	ENST0000039432	E17	1836 (149		C	C -> T (het)	36% (193)	[40% (65) / 34% (128)]	S (497	COSM4002932	c.14914C>T	p.Pro4972Ser	distinct
158	FAT1	ENST0000044180	E2	1860 (184		C	C -> G (het)	30% (216)	[30% (108) / 30% (108)]	L (614	COSM4003038	c.1842C>G	p.Phe614Leu	distinct
159	FAT4	ENST0000039432	E9	2426 (987		C	G -> A (het)	63% (385)	[61% (169) / 65% (216)]	G (329	rs115219562 (1	c.9870G>A	p.Gly3290=	distinct
160	FAT4	ENST0000039432	E1	2433 (242		C	C -> T (homo)	100% (968)	[100% (592) / 100% (376)]	V (807	COSM3760371	c.2420C>T	p.Ala807Val	distinct
161	FAT1	ENST0000044180	E2	2602 (258		C	G -> C (het)	33% (376)	[33% (212) / 33% (164)]	> L (862	COSM4003037	c.2584G>C	p.Val862Leu	distinct
162	SETD2	ENST0000040979	E3	2617 (270		C	G -> C (homo)	100% (961)	[100% (526) / 100% (435)]	Q (902	rs58906143 (10	c.2704G>C	p.Glu902Gln	distinct
163	FAT4	ENST0000039432	E1	2824 (281		C	G -> T (het)	26% (284)	[26% (136) / 26% (148)]	N (937	rs112454576 (1	c.2811G>T	p.Lys937Asn	distinct
164	FAT4	ENST0000039432	E1	2957 (294		C	T -> C (homo)	100% (667)	[100% (345) / 100% (322)]	L (982	rs2940779 (100	c.2944T>C	p.Leu982=	distinct
165	FAT1	ENST0000044180	E2	3208 (319		C	A -> G (homo)	100% (959)	[100% (479) / 100% (480)]	G (106	COSM4003035	c.3190A>G	p.Arg1064Gly	distinct
166	FAT1	ENST0000044180	E10	3988 (879		C	A -> T (het)	71% (575)	[70% (256) / 73% (319)]	L (293	rs1280098 (100	c.8798A>T	p.Gln2933Leu	distinct
167	FAT1	ENST0000044180	E10	3988 (879		C	A -> C (het)	29% (231)	[30% (111) / 27% (120)]	P (293	COSM4005727	c.8798A>C	p.Gln2933Pro	distinct
168	FAT4	ENST0000039432	E9	4038 (114		C	A -> G (het)	64% (611)	[63% (290) / 65% (321)]	E (382	rs17009684 (10	c.11482A>G	p.Lys3828Glu	distinct
169	FAT4	ENST0000039432	E9	4174 (116		C	G -> A (homo)	100% (916)	[100% (454) / 100% (462)]	N (387	rs12650153 (10	c.11618G>A	p.Ser3873Asn	distinct
170	FAT4	ENST0000039432	E1	4318 (430		C	C -> T (homo)	100% (1097)	[100% (536) / 100% (561)]	I (1435	rs2710555 (100	c.4305C>T	p.Ile1435=	distinct
171	LRP1B	ENST0000038948	E55	+3	[chr2:	C	A -> G (het)	24% (72)	[27% (20) / 23% (52)]		rs117225004 (1	c.8850+3A>G		distinct
172	ROBO1	ENST0000046423	E26	+4	[chr3:	C	T -> C (homo)	100% (679)	[100% (303) / 100% (376)]		rs7636043 (100	c.3875+4T>C		distinct
173	LRP1B	ENST0000038948	E33	+5	[chr2:	C	A -> G (homo)	100% (380)	[100% (144) / 100% (236)]		rs996361 (1000	c.5500+5A>G		distinct
174	PBRM1	ENST0000039483	E29	+6 / 3bp	[	I	GAG (homo)	88% (469)	[91% (166) / 87% (303)]		rs71084187 (db	.4576+5_4576+6insGAG		distinct
175	SF1	ENST0000037739	E6	+6	[chr11	C	C -> T (het)	48% (736)	[52% (424) / 42% (312)]		rs148875917 (1	c.663+6C>T		distinct
176	GATA6	ENST0000026921	E6	+7	[chr18	C	A -> G (homo)	100% (219)	[100% (53) / 100% (166)]		COSM4000377	c.1620+7A>G		distinct
177	LRP1B	ENST0000038948	E19	+10	[chr2	C	A -> C (homo)	100% (217)	[100% (78) / 100% (139)]		rs1429348 (100	c.2968+10A>C		distinct
178	LRP1B	ENST0000038948	E49	+12	[chr2	C	A -> T (het)	63% (347)	[62% (101) / 64% (246)]		rs13404083 (10	c.8026+12A>T		distinct

179	LMNA	ENST0000036830	E4	+13 [chr1	C	G -> T (het)	61% (576)	[57% (227) / 64% (349)]		rs11264444 (10	c.810+13G>T			distinct
180	LRP1B	ENST0000038948	E30	+14 / 1bp	I	A (het)	87% (451)	[79% (152) / 92% (299)]		rs78477398 (10	.5114+13_5114+14insA			distinct
181	LRP1B	ENST0000038948	E34	+15 [chr2	C	A -> G (het)	71% (411)	[72% (155) / 70% (256)]		rs13382825 (10	c.5626+15A>G			distinct
182	MYCBP2	ENST0000035733	E42	+15 / 1bp	D	T (homo)	95% (242)	[100% (33) / 95% (209)]		rs397840781 (d	c.6226+15delT			distinct
183	KMT2C	ENST0000026218	E7	+15 [chr7	C	G -> C (het)	24% (198)	[23% (59) / 24% (139)]		rs201635031 (d	c.1012+15G>C			distinct
184	CHD1	ENST0000028404	E13	+16 [chr5	C	A -> G (homo)	100% (337)	[100% (93) / 100% (244)]		rs17166428 (10	c.1991+16A>G			distinct
185	SLIT2	ENST0000050415	E28	+16 [chr4	C	A -> G (het)	23% (102)	[25% (41) / 22% (61)]		rs12506323 (10	c.2948+16A>G			distinct
186	MARK2	ENST0000040201	E11	+16 [chr1	C	C -> T (het)	51% (405)	[50% (114) / 51% (291)]		rs224174 (1000	c.1101+16C>T			distinct
187	SMARCA4	ENST0000045071	E31	+16 [chr1	C	G -> T (homo)	100% (565)	[100% (282) / 100% (283)]		rs151265814 (1	c.4675+16G>T			distinct
188	NARF	ENST0000030979	E3	+17 [chr1	C	A -> T (het)	74% (577)	[73% (149) / 74% (428)]		rs28401416 (10	c.252+17A>T			distinct
189	ROBO3	ENST0000039780	E4	+24 [chr1	C	T -> C (homo)	100% (793)	[100% (417) / 100% (376)]		rs4936957 (100	c.766+24T>C			distinct
190	SF1	ENST0000037739	E6	+32 [chr1	C	A -> G (homo)	100% (1454)	[100% (607) / 100% (847)]		rs484886 (1000	c.663+32A>G			distinct
191	POLR3A	ENST0000037237	E22	+42 [chr1	C	T -> C (het)	59% (449)	[58% (226) / 60% (223)]		rs3815891 (100	c.2988+42T>C			distinct
192	BRD2	ENST0000044908	E9	+663 / 4bp	I (Dup)	GTTT (het)	56% (76)	[79% (55) / 32% (21)]			c.1700+659_1700+662dupGTTT			distinct
193	BRD2	ENST0000044908	E9	+672 [chr	C	A -> T (het)	84% (97)	[85% (52) / 82% (45)]		rs2395380 (100	c.1700+672A>T			distinct
194	BRD2	ENST0000044908	E9	+689 [chr	C	G -> T (homo)	95% (109)	[91% (52) / 98% (57)]		rs2082260 (100	c.1700+689G>T			distinct
195	BRD2	ENST0000044908	E9	+698 / 1bp	I (Dup)	T (het)	40% (47)	[40% (20) / 40% (27)]			c.1700+697dupT			distinct
196	SLIT2	ENST0000050415	E1	+1105 [ch	C	G -> A (het)	28% (334)	[29% (137) / 27% (197)]		rs1323066 (100	c.179+1105G>A			distinct
197	SMAD4	ENST0000034298	E4	+2088 [ch	C	G -> C (homo)	100% (89)	[100% (42) / 100% (47)]		rs7229678 (100	c.454+2088G>C		2/1 of 3	distinct
198	SMAD3	ENST0000032736	E1	+32698 / 1	D	T (homo)	98% (1802)	[98% (797) / 97% (1005)]			c.206+32698delT			distinct
199	ATM	ENST0000027861	E1	275 [chr1	C	G -> A (homo)	100% (280)	[100% (140) / 100% (140)]	' UTR	rs189037 (1000	c.-111G>A		1/0 DP	distinct
200	BRCA1	ENST0000035765	E10	1942 (261	C	C -> T (homo)	100% (1429)	[100% (679) / 100% (750)]	L (871	COSM148278 (	c.2612C>T	p.F	32/ DP, POLY	distinct
201	ASCC3	ENST0000036916	E12	33 (1935)	C	C -> T (homo)	100% (525)	[100% (343) / 99% (182)]	L (645	rs41288423 (10	c.1935C>T	p.L	88/ Ident SNI	distinct
202	MMACHC	ENST0000040106	E3	45 (321) [	C	G -> A (het)	29% (242)	[28% (107) / 31% (135)]	V (107	rs2275276 (100	c.321G>A	p.V	77/ Ident-SNI	distinct
203	TPH2	ENST0000033385	E9	57 (1125)	C	A -> T (homo)	100% (895)	[100% (523) / 100% (372)]	A (375	COSM3753489	c.1125A>T	p.A	162 Ident-SNI	distinct
204	EDNRB	ENST0000044657	E4	30 (831) [	C	A -> G (homo)	100% (499)	[100% (286) / 100% (213)]	L (277	COSM3753750	c.831A>G	p.L	156 Ident-SNI	distinct
205	GABRG3	ENST0000033374	E8	98 (963) [	C	C -> T (het)	37% (629)	[37% (366) / 36% (263)]	T (321	rs140679 (1000	c.963C>T	p.T	100 Ident-SNI	distinct
206	GABRG3	ENST0000033374	E8	98 (963) [	C	C -> T (het)	37% (629)	[37% (366) / 36% (263)]	T (321	rs140679 (1000	c.963C>T	p.T	100 Ident-SNI	distinct
207	ATP13A4	ENST0000039244	E6	10 (543) [	C	A -> G (het)	29% (249)	[30% (163) / 28% (86)]	M (181	COSM3759936	c.543A>G	p.Il	55/ Ident-SNI	distinct
208	WDFY3	ENST0000032236	E6	32 (336) [	C	A -> G (het)	66% (523)	[67% (280) / 65% (243)]	L (112	COSM4003176	c.336A>G	p.L	56/ Ident-SNI	distinct
209	MUT	ENST0000027481	E3	251 (636)	C	G -> A (homo)	100% (435)	[100% (226) / 99% (209)]	K (212	rs2229384 (100	c.636G>A	p.L	151 Ident-SNI	distinct
210	MPDZ	ENST0000031921	E25	157 (3609)	C	A -> G (het)	29% (168)	[31% (69) / 27% (99)]	K (120	rs10756457 (10	c.3609A>G	p.L	47/ Ident-SNI	distinct
211	LMNA	ENST0000036830	E9	-41 [chr1	C	C -> T (homo)	100% (1131)	[100% (705) / 99% (426)]		rs553016 (1000	c.1489-41C>T		2/5 POLY	distinct
212	SMARCA2	ENST0000034972	E33	-20 [chr9	C	T -> C (homo)	100% (123)	[100% (82) / 100% (41)]		rs3818384 (100	c.4595-20T>C		0/2 POLY	distinct
213	CHD6	ENST0000037323	E14	-19 [chr2	C	G -> A (homo)	100% (974)	[100% (600) / 100% (374)]		rs4812516 (100	c.1858-19G>A		0/1 POLY	distinct
214	PIK3CA	ENST0000026396	E6	-17 [chr3	C	C -> A (homo)	100% (785)	[100% (497) / 100% (288)]		rs2699896 (100	c.1060-17C>A		67/ POLY	distinct
215	MEN1	ENST0000031204	E2	-16 [chr1	C	C -> G (het)	56% (98)	[56% (96) / 50% (2)]		rs509606 (1000	c.-23-16C>G		1/5 POLY	forced,distinct
216	BRAF	ENST0000028860	E18	-16 [chr7	C	C -> T (het)	82% (14)	[100% (10) / 57% (4)]		rs368721021 (C	c.2128-16C>T		1/1 POLY	distinct
217	BRCA2	ENST0000054445	E17	-14 [chr1	C	T -> C (homo)	100% (469)	[100% (365) / 100% (104)]		rs9534262 (100	c.7806-14T>C		85/ POLY	distinct
218	PIK3CA	ENST0000026396	E12	-13 [chr3	C	T -> C (het)	52% (267)	[57% (200) / 41% (67)]		rs41273619 (10	c.1747-13T>C		0/1 POLY	distinct
219	ATM	ENST0000027861	E24	-12 / 1bp	I (Dup)	A (het)	85% (130)	[80% (89) / 98% (41)]		ClinVar ben (Mu	c.3403-13dupA		38/ POLY	distinct
220	JAG1	ENST0000025495	E6	10 (765) [	C	C -> T (het)	35% (310)	[35% (144) / 35% (166)]	Y (255	COSM3758428	c.765C>T	p.T	5/7 POLY	distinct
221	KMT2D	ENST0000030106	E11	29 (2826)	C	C -> T (homo)	100% (833)	[100% (421) / 100% (412)]	I (942)	COSM3753294	c.2826C>T	p.Il	3/1 POLY	distinct
222	ATM	ENST0000027861	E40	30 (5948)	C	A -> G (homo)	100% (97)	[100% (44) / 100% (53)]	S (198	rs659243 (1000	c.5948A>G	p.A	272 POLY	distinct

223	SMARCA2	ENST0000034972	E2	32 [chr9:	C	G -> A (het)	58% (199)	[59% (127) / 58% (72)]	' UTR	rs10964468 (10	c.-5G>A		0/3	POLY	distinct
224	KRAS	ENST0000025607	E5	33 (483) [	C	G -> A (homo)	100% (947)	[100% (422) / 100% (525)]	> R (161	rs4362222 (100	c.483G>A	p.A	79/	POLY	distinct
225	APC	ENST0000025743	E12	50 (1458)	C	T -> C (homo)	100% (723)	[100% (337) / 100% (386)]	> Y (486	COSM1432175	c.1458T>C	p.T	3/2	POLY	distinct
226	LMNA	ENST0000036830	E5	51 (861) [	C	T -> C (het)	32% (339)	[30% (143) / 34% (196)]	> A (287	rs538089 (1000	c.861T>C	p.A	2/8	POLY	distinct
227	FGFR2	ENST0000035848	E6	72 (696) [	C	A -> G (homo)	100% (1149)	[100% (490) / 100% (659)]	> V (232	rs1047100 (100	c.696A>G	p.V	0/1	POLY	distinct
228	FGFR2	ENST0000047885	E4	94 [chr10	C	A -> G (homo)	100% (668)	[100% (466) / 100% (202)]	' UTR	rs2981437 (100	c.-109A>G		1/0	POLY	distinct
229	KMT2D	ENST0000030106	E39	96 (10836)	C	G -> A (homo)	100% (827)	[100% (365) / 100% (462)]	Q (361	COSM431202 (	c.10836G>A	p.G	3/1	POLY	distinct
230	SMAD3	ENST0000032736	E2	103 (309)	C	A -> G (homo)	100% (1004)	[100% (623) / 100% (381)]	> L (103)	rs1065080 (100	c.309A>G	p.L	12/	POLY	distinct
231	LMNA	ENST0000036830	E12	106 [chr1	C	G -> C (homo)	100% (503)	[100% (191) / 100% (312)]	' UTR	rs7339 (1000Ge	c.*79G>C		2/7	POLY	distinct
232	MEN1	ENST0000031204	E9	114 (1299)	C	T -> C (homo)	100% (1269)	[100% (670) / 100% (599)]	> H (433	rs540012 (1000	c.1299T>C	p.H	20/	POLY	distinct
233	TP53	ENST0000026930	E4	119 (215)	C	C -> G (homo)	100% (735)	[100% (313) / 100% (422)]	> R (72)	COSM3766192	c.215C>G	p.P	14	POLY	distinct
234	ARID1B	ENST0000034608	E6	135 (2172)	C	G -> A (homo)	100% (969)	[100% (377) / 100% (592)]	> A (724	COSM1487405	c.2172G>A	p.A	4/1	POLY	distinct
235	BCORL1	ENST0000054005	E3	154 (331)	C	T -> C (homo)	100% (698)	[100% (404) / 100% (294)]	> L (111)	rs4830173 (100	c.331T>C	p.P	1/0	POLY	distinct
236	KMT2D	ENST0000030106	E10	168 (1426)	C	G -> A (homo)	98% (511)	[94% (127) / 100% (384)]	> T (476	rs1064210 (100	c.1426G>A	p.A	0/3	POLY	distinct
237	KMT2D	ENST0000030106	E10	168 (1426)	C	G -> A (homo)	98% (511)	[94% (127) / 100% (384)]	> T (476	rs1064210 (100	c.1426G>A	p.A	0/3	POLY	distinct
238	LMNA	ENST0000036830	E7	181 (1338)	C	T -> C (homo)	100% (216)	[100% (80) / 100% (136)]	> D (446	rs505058 (1000	c.1338T>C	p.A	2/8	POLY	distinct
239	LMNA	ENST0000036830	E7	181 (1338)	C	T -> C (homo)	100% (216)	[100% (80) / 100% (136)]	> D (446	rs505058 (1000	c.1338T>C	p.A	2/8	POLY	distinct
240	SMARCA2	ENST0000034972	E25	216 (3672)	C	G -> A (homo)	100% (537)	[100% (269) / 100% (268)]	E (1224	COSM3763856	c.3672G>A	p.G	0/4	POLY	distinct
241	JAG1	ENST0000025495	E26	218 (3417)	C	T -> C (homo)	100% (866)	[100% (403) / 100% (463)]	Y (1139	COSM3758426	c.3417T>C	p.T	5/7	POLY	distinct
242	CHD1	ENST0000028404	E35	263..265 (	D	CCT (homo)	97% (1467)	[97% (808) / 96% (659)]	(1684	COSM327350 (	c.5050_5052delCCT	p.P	0/1	POLY	distinct
243	MEN1	ENST0000031204	E10	271 (1621)	C	A -> G (homo)	100% (1341)	[100% (779) / 100% (562)]	> A (541	COSM255213 (	c.1621A>G	p.T	20/	POLY	distinct
244	SMAD3	ENST0000032736	E1	296 [chr1	C	G -> A (het)	26% (66)	[21% (38) / 36% (28)]	' UTR	rs1061427 (100	c.-15G>A		0/8	POLY	distinct
245	RNF43	ENST0000040797	E9	300 (1252)	C	C -> A (homo)	100% (2000)	[100% (1063) / 100% (937)]	M (418	COSM4130449	c.1252C>A	p.L	13/	POLY	distinct
246	NF2	ENST0000033864	E1	332 [chr2	C	G -> C (homo)	95% (158)	[95% (154) / 100% (4)]	' UTR	rs1800540 (100	c.-110G>C		12/	POLY	forced,distinct
247	CASR	ENST0000049013	E7	512 (2244)	C	G -> C (homo)	100% (2197)	[100% (1122) / 100% (1075)]	> P (748	rs2036400 (100	c.2244G>C	p.P	3/1	POLY	distinct
248	KMT2C	ENST0000026218	E14	635 (2448)	I (Dup)	A (het)	27% (195)	[29% (68) / 27% (127)]	816 (R	rs150073007 (d	c.2447dupA	p.T	0/1	POLY	distinct
249	KMT2D	ENST0000030106	E31	1245 (747	C	G -> T (homo)	100% (1035)	[100% (679) / 99% (356)]	G (249	COSM3722550	c.7479G>T	p.G	3/1	POLY	distinct
250	KMT2D	ENST0000030106	E31	1245 (747	C	G -> T (homo)	100% (1035)	[100% (679) / 99% (356)]	G (249	COSM3722550	c.7479G>T	p.G	3/1	POLY	distinct
251	CASR	ENST0000049013	E7	1299 (303	C	G -> C (homo)	100% (1268)	[100% (577) / 100% (691)]	Q (101	rs1801726 (100	c.3031G>C	p.G	3/1	POLY	distinct
252	CASR	ENST0000049013	E7	1565 [chr	C	A -> T (homo)	100% (623)	[100% (159) / 100% (464)]	' UTR	rs4677948 (100	c.*60A>T		3/1	POLY	distinct
253	BRCA2	ENST0000054445	E11	2654 (456	C	A -> G (homo)	100% (544)	[100% (280) / 100% (264)]	L (1521	rs206075 (1000	c.4563A>G	p.L	657	POLY	distinct
254	SETD2	ENST0000040979	E3	3378 (346	C	T -> C (homo)	100% (1061)	[100% (579) / 100% (482)]	N (115	rs6767907 (100	c.3465T>C	p.A	1/6	POLY	distinct
255	APC	ENST0000025743	E16	3507 (546	C	T -> A (homo)	100% (345)	[100% (152) / 100% (193)]	D (182	COSM3760871	c.5465T>A	p.V	4/1	POLY	distinct
256	BRCA2	ENST0000054445	E11	4604 (651	C	G -> C (homo)	100% (335)	[100% (214) / 100% (121)]	V (217	COSM4147689	c.6513G>C	p.V	301	POLY	distinct
257	CASR	ENST0000049013	E7	+15 / 1bp	I (Dup)	A (het)	29% (22)	[0% (0) / 29% (22)]		rs33974189 (Mu	c.*188dupA		0/2	POLY	forced,distinct
258	LMNA	ENST0000036830	E6	+16 [chr1	C	G -> A (het)	37% (422)	[35% (184) / 38% (238)]		rs534807 (1000	c.1157+16G>A		2/8	POLY	distinct
259	LMNA	ENST0000036830	E6	+16 [chr1	C	G -> A (het)	37% (422)	[35% (184) / 38% (238)]		rs534807 (1000	c.1157+16G>A		2/8	POLY	distinct
260	CASR	ENST0000049013	E3	+19 [chr3	C	G -> A (homo)	99% (373)	[100% (10) / 99% (363)]		rs9869985 (100	c.492+19G>A		3/1	POLY	forced,distinct
261	TP53	ENST0000026930	E2	+38 [chr1	C	C -> G (het)	93% (240)	[61% (30) / 100% (210)]		rs1642785 (100	c.74+38C>G		117	POLY	distinct
262	BRCA1	ENST0000035765	E1	+101 [chr	C	C -> G (homo)	99% (307)	[100% (230) / 99% (77)]		rs799905 (1000	c.-20+101C>G		1/0	POLY	distinct
263	KDM6A	ENST0000037796	E18	-5 / 1bp [	D	T (het)	45% (35)	[42% (16) / 48% (19)]		ExAC, ClinVar (I	c.2703-5delT		0/1	POLY (sel	distinct
264	RNF43	ENST0000040797	E2	524 (139)	C	A -> G (het)	22% (396)	[22% (188) / 21% (208)]	> V (47)	rs3744093 (100	c.139A>G	p.Il	14/	Poly	distinct
265	SMARCA2	ENST0000034972	E4	350..352 (	D	GCA (het)	36% (102)	[36% (46) / 36% (56)]	Q (235..236)		c.705_707delGCA	p.G	0/4	Sequenza	distinct
266	BRCA2	ENST0000054445	E14	390 (7397)	C	T -> C (homo)	100% (282)	[100% (91) / 100% (191)]	A (246	rs169547 (1000	c.7397T>C	p.V	302	Wildtyp	distinct
