# Supplementary figures and images for: The Gain-of-Function p53 R248W Mutant Promotes Migration by STAT3 Deregulation in Human Pancreatic Cancer Cells
Source: Front Oncol. 2021 Jun 11;11:642603. doi: 10.3389/fonc.2021.642603 (PMC8226097; doi:10.3389/fonc.2021.642603)

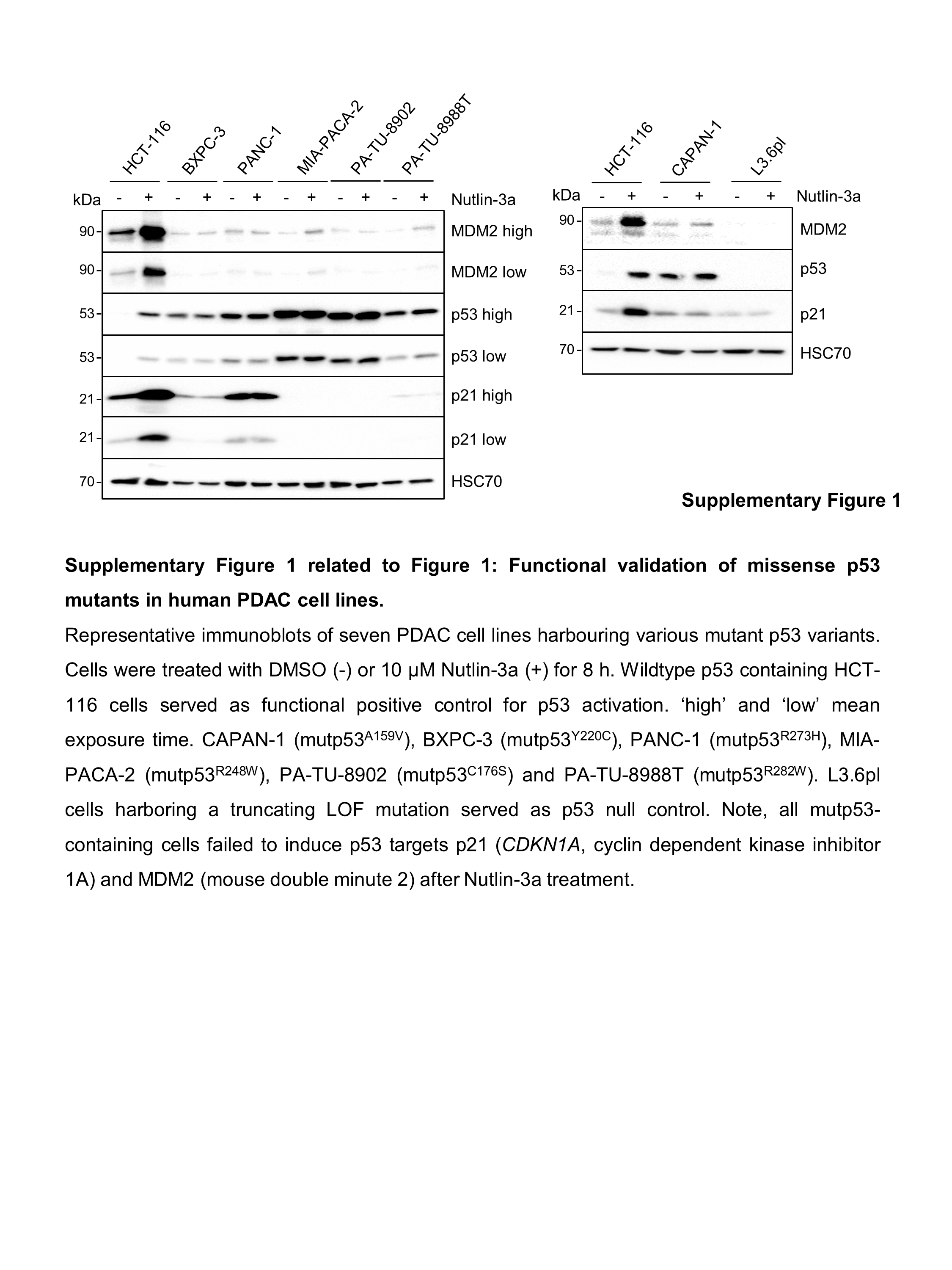

Supplement: Supplementary file 2 [file Image_1.tif]

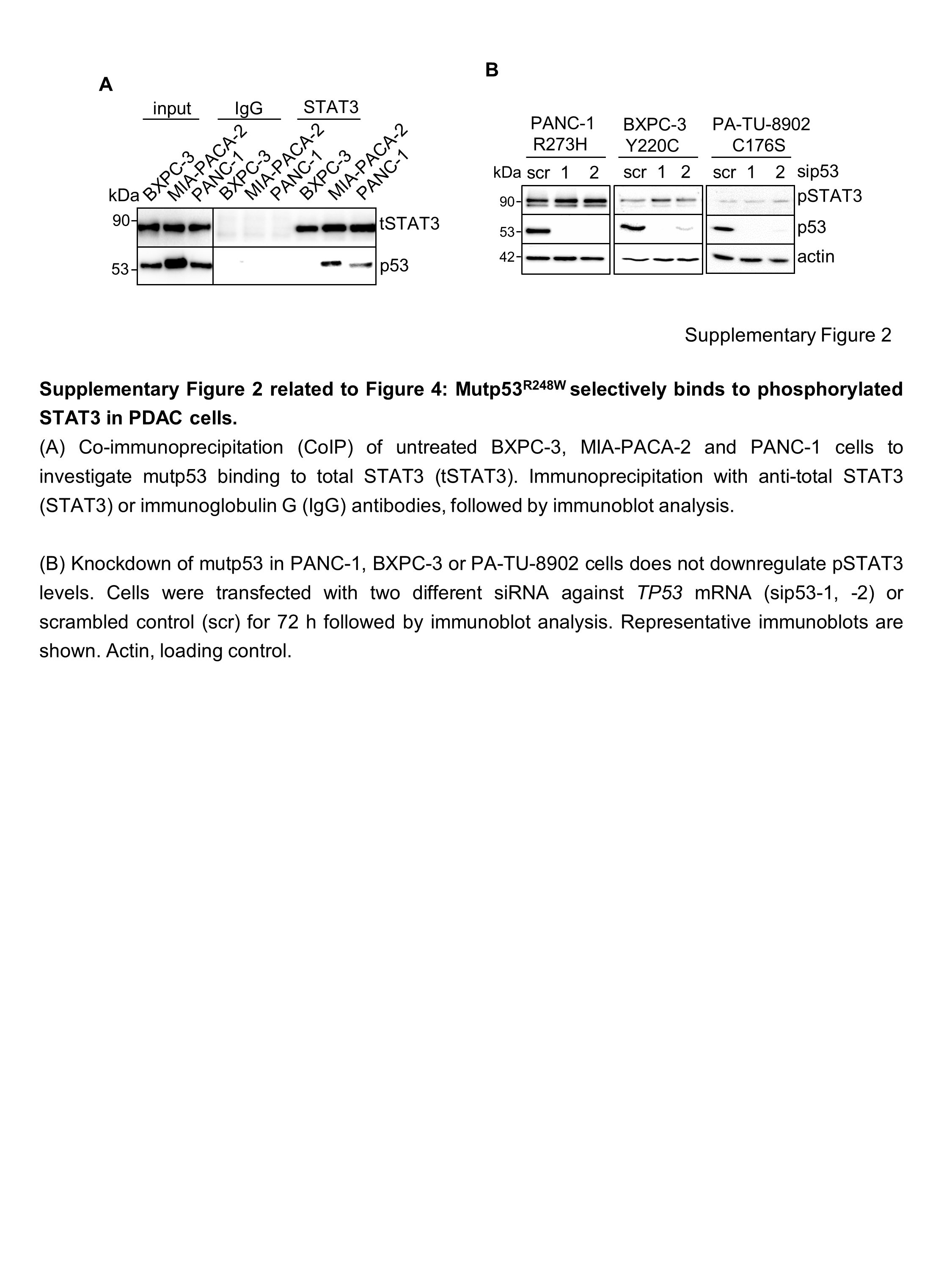

Supplement: Supplementary file 3 [file Image_2.tif]

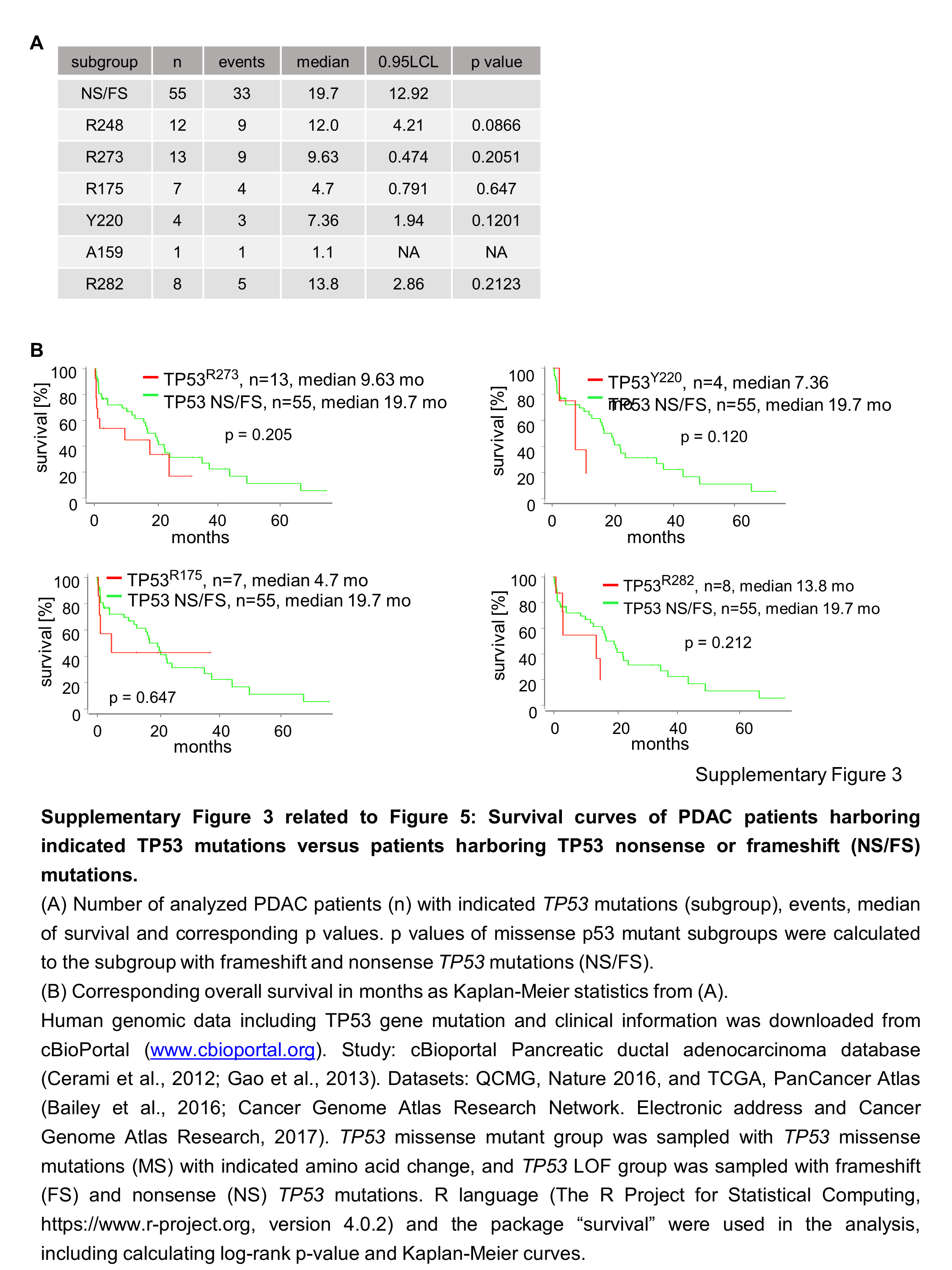

Supplement: Supplementary file 4 [file Image_3.tif]
