# Supplementary material for: The Gain-of-Function p53 R248W Mutant Promotes Migration by STAT3 Deregulation in Human Pancreatic Cancer Cells
Source: Front Oncol. 2021 Jun 11;11:642603. doi: 10.3389/fonc.2021.642603 (PMC8226097; doi:10.3389/fonc.2021.642603)
Supplement: Supplementary file 5 [file Table_1.pdf]

Supplementary Table 1: Reagents and Resources

REAGENT or RESOURCE	SOURCE	IDENTIFIER		
**Antibodies**			**WB**	**Co-IP**
Rabbit monoclonal anti-Akt [D9E]	Cell Signaling	9272, RRID:AB_329827	1:1,000	
Mouse polyclonal anti-beta-Actin	Abcam	ab6276, RRID:AB_2223210	1:10,000	
Rabbit polyclonal anti-beta-Actin	Abcam	ab8227, RRID:AB_2305186	1:10,000	
Mouse monoclonal anti-HSC70 [B-6]	Santa Cruz	sc-7298, RRID:AB_627761	1:5,000	
Mouse monoclonal anti-MDM2 (Ab-1) [IF-2]	Calbiochem®/Millipore	OP46, RRID:AB_2335867	1:300	
Rabbit monoclonal anti-p21 Waf1/Cip1 [12D1]	Cell Signaling	2947, RRID:AB_823586	1:1,000	
Mouse monoclonal anti-p53 [DO-1]	Santa Cruz	sc-126, RRID:AB_628082	1:10,000	
Mouse monoclonal anti-p53 [DO-1], HRP conjugated	Santa Cruz	sc-126 HRP, RRID:AB_628082	1:1,000	3µg
Rabbit monoclonal anti-phospho-Y705 STAT3 [EP2147Y]	Abcam	ab76315, RRID:AB_1658549	1:2,000	3µg
Rabbit polyclonal anti-STAT3	Santa Cruz	sc-482, RRID:AB_632440	1:1,000	
Rabbit monoclonal anti-STAT3 [79D7]	Cell Signaling	4904, RRID:AB_331269	1:1,000	3µg
Rabbit monoclonal anti-IgG [EPR25A]	Abcam	ab172730, RRID:AB_2687931		3µg
goat anti-rabbit IgG-HRP	Santa Cruz	sc-2004, RRID:AB_631746	1:10,000	
goat anti-mouse IgG-HRP	Santa Cruz	sc-2005, RRID:AB_631736	1:10,000	
**Chemicals, Peptides and Recombinant Proteins**				
BCA protein assay	Pierce	23227		
CellTiter-Glo® Luminescent Cell Viability Assay	Promega	G7571		
Clarity Max™ Western ECL Substrate	BioRad	1705062		
cOmplete™ mini protease inhibitor cocktail	Roche	11836170001		
Crystal violet (C.I. 42555)	Roth	T123.1		
Cycloheximide	Sigma-Aldrich	C7698		
Dimethyl sulfoxide (DMSO) Cell culture grade	AppliChem	A3672		
EDTA	Roth	8040.1		
Ethanol absolut	Chemsolute®/Th.Geyer	2246		
Ganetespib	Provided by Synta Pharmaceuticals	N/A		
Imidazol	Roth	899.2		
Immobilion Western chemiluminescent HRP substrate	Millipore/Merck	WBKLS0500		
InSolution™ MG-132	Calbiochem®/M	474791		

	erck	
Interleukin-6	Immunotools	11340064
Methanol	Roth	8388.3
Milk powder	Roth	T145.4
NaCl	Roth	3957.2
NaF	AppliChem	A0401
Nitrocellulose membranes	Amersham	GE10600001
Nonidet™ P 40 Substitute	Sigma-Aldrich	74385
Nutlin-3a	BOC Sciences	B0084-425358
Onalespib	Selleckchem	S1163
Oncostatin M (209a.a)	Immunotools	11344223
Protein G Sepharose 4 Fast Flow	GE Healthcare	17061805
SDS	Roth	CN30.3
Sodium deoxycholate	Sigma-Aldrich	30970
Sodium orthovanadate	Sigma-Aldrich	S6508
Stattic	Santa Cruz	sc-202818
SuperSignal™ West Femto Maximum Sensitivity Substrate	ThermoFisher Scientific	34095
Tris-HCl	Roth	4855.3
TritonX-100	AppliChem	A1388
Tween-20	AppliChem	A4974
**Reagents for Cell culture**		
DMEM	Gibco	31600091
FBS	Merck	S0615
L-Glutamine	Gibco	25030123
Mycoplasma Detection Kit	Lonza	LT07-318
Penicillin-Streptomycin	Gibco	15140122
RPMI 1640	Gibco	42401042
Lipofectamine™ 3000 Transfection Reagent	Invitrogen	L3000015
Lipofectamine™ 2000 Transfection Reagent	Invitrogen	11668019
TC treated 6 well plates	Sarstedt	83.3920
TC treated 96 well plates	Corning	3903
Falcon® Permeable Support for 24-well Plate with 8.0 µm Transparent PET Membrane	Corning	353097
Falcon® 24-well TC-treated Cell Polystyrene Permeable Support Companion Plate	Corning	353504

Experimental models: Cell lines

			96 well	Transwell insert	6 well cmp	6 well transfection
L3.6pl	RRID:CVCL_0384	PMID: 23917223; PMID: 10935470	5,000	-	150,000	-
MIA-PACA-2	DSMZ, RRID:CVCL_0428	ACC 733	3,000	70,000	120,000	80,000
PANC-1	ATCC, RRID:CVCL_0480	CRL-1469	5,000	50,000	200,000	100,000

PA-TU-8988T	DSMZ, RRID:CVCL_1847	ACC 162	4,000	70,000	150,000	90,000
PA-TU-8902	DSMZ, RRID:CVCL_1845	ACC 179	4,000	70,000	150,000	100,000
BXPC-3	ATCC, RRID:CVCL_0186	CRL-1687	5,000	100,000	200,000	100,000
CAPAN-1	ATCC, RRID:CVCL_0237	HTB-79	10,000	-	300,000	-
					cmp, compound	

**Oligonucleotides and Recombinant DNA**		
siRNA Silencer™ Select Negative Control No. 2 siRNA (scr)	Invitrogen	4390847
siRNA p53 Silencer™ Select No.1	ThermoFisher Scientific	4390824, siRNA ID s605
siRNA p53 Silencer™ Select No.2	ThermoFisher Scientific	4390824, siRNA ID s606
siRNA p53 Silencer™ Select No.3	ThermoFisher Scientific	AM51331, siRNA ID 106141
siRNA STAT3 Silencer™ Select No.1	ThermoFisher Scientific	4390824, siRNA ID s743
siRNA STAT3 Silencer™ Select No.2	ThermoFisher Scientific	4390824, siRNA ID s744
**Software and Algorithms**		
ImageJ software	Open source	https://imagej.net/Welcome
Image Lab™ Software	Biorad	http://www.bio-rad.com/de-de/product/image-lab-software
ZEN	Zeiss	https://www.zeiss.de/mikroskopie/produkte/mikroskopsoftware/zen.html
Image Lab™ Software	Biorad	http://www.bio-rad.com/de-de/product/image-lab-software
Adobe Photoshop Software	Adobe	https://www.adobe.com/de/creativecloud/plans.html
Celigo Imaging Cytometer	Nexcelom Bioscience	https://www.nexcelom.com/nexcelom-products/cellometer-and-celigo-image-cytometers/celigo-imaging-cytometer/
